# Analysis of DNA polymerase ν function in meiotic recombination, immunoglobulin class-switching, and DNA damage tolerance

**DOI:** 10.1371/journal.pgen.1006818

**Published:** 2017-06-01

**Authors:** Kei-ichi Takata, Shelley Reh, Matthew J. Yousefzadeh, Maciej J. Zelazowski, Sarita Bhetawal, David Trono, Megan G. Lowery, Maria Sandoval, Yoko Takata, Yue Lu, Kevin Lin, Jianjun Shen, Donna F. Kusewitt, Kevin M. McBride, Francesca Cole, Richard D. Wood

**Affiliations:** 1 Department of Epigenetics and Molecular Carcinogenesis, The University of Texas MD Anderson Cancer Center, Smithville, Texas, United States of America; 2 The University of Texas MD Anderson Cancer Center UT Health Graduate School of Biomedical Sciences, Houston, Texas, United States of America; Cornell University, UNITED STATES

## Abstract

DNA polymerase ν (pol ν), encoded by the *POLN* gene, is an A-family DNA polymerase in vertebrates and some other animal lineages. Here we report an in-depth analysis of pol ν–defective mice and human cells. *POLN* is very weakly expressed in most tissues, with the highest relative expression in testis. We constructed multiple mouse models for *Poln* disruption and detected no anatomic abnormalities, alterations in lifespan, or changed causes of mortality. Mice with inactive *Poln* are fertile and have normal testis morphology. However, pol ν–disrupted mice have a modestly reduced crossover frequency at a meiotic recombination hot spot harboring insertion/deletion polymorphisms. These polymorphisms are suggested to generate a looped-out primer and a hairpin structure during recombination, substrates on which pol ν can operate. Pol ν-defective mice had no alteration in DNA end-joining during immunoglobulin class-switching, in contrast to animals defective in the related DNA polymerase θ (pol θ). We examined the response to DNA crosslinking agents, as purified pol ν has some ability to bypass major groove peptide adducts and residues of DNA crosslink repair. Inactivation of *Poln* in mouse embryonic fibroblasts did not alter cellular sensitivity to mitomycin C, cisplatin, or aldehydes. Depletion of *POLN* from human cells with shRNA or siRNA did not change cellular sensitivity to mitomycin C or alter the frequency of mitomycin C-induced radial chromosomes. Our results suggest a function of pol ν in meiotic homologous recombination in processing specific substrates. The restricted and more recent evolutionary appearance of pol ν (in comparison to pol θ) supports such a specialized role.

## Author summary

The work described here fills a current gap in the study of the 16 known DNA polymerases in vertebrate genomes. Until now, experiments with genetically disrupted mice have been reported for all but pol ν, encoded by the *POLN* gene. To intensively analyze the role of mammalian pol ν we generated multiple *Poln*-deficient murine models. We discovered that *Poln* is uniquely upregulated during testicular development and that it is enriched in spermatocytes. This, and phylogenetic analysis indicate a testis-specific function. We observed a modest reduction in meiotic recombination at a recombination hotspot in *Poln*-deficient mice. Pol ν has been suggested to function in DNA crosslink repair. However, we found no increased DNA crosslink sensitivity in *Poln*-deficient mice or *POLN*-depleted human cells. This is a major difference from some previous findings, and we support our conclusion by multiple experimental approaches, and by the very low or absent expression of functional pol ν in mammalian somatic cells. The present work represents the first description and comprehensive analysis of mice deficient in pol ν, and the first thorough phenotypic analysis in human cells.

## Introduction

In mammalian cells, a diverse group of DNA polymerases carry out genomic DNA replication and genome maintenance. These include the core enzymes for semi-conservative DNA replication (pols α, δ, ε and telomerase), base excision repair (pol β), mitochondrial DNA replication and repair (pol γ and Primpol), non-homologous end-joining and immunological diversity (pols λ, μ, θ and terminal-deoxynucleotidyl transferase), and DNA damage tolerance by translesion synthesis (η, ι, κ, ζ, and Rev1). Some of these enzymes have roles in more than one pathway of DNA processing [1, 2]. The consequences of genetic disruption of an individual DNA polymerase vary widely, ranging from embryonic lethality to no discernable phenotype.

Pol ν, encoded by the *POLN* gene in mammalian cells, is not assigned in the preceding list, and it is the only identified polymerase for which an analysis of knockout animals remains to be described. The catalytic domain of pol ν belongs to the A-family of DNA polymerases, and is related to the pol domain of pol θ (encoded by mammalian *POLQ* / Drosophila *Mus308*) [3–6]. Pol θ participates in a pathway of DNA double-strand break repair by alternative end joining [7, 8], and consequently defects in *Mus308* or *POLQ* confer hypersensitivity to various DNA damaging agents [8–10]. The function of pol ν is currently uncertain, but several roles have been suggested. For example, it was reported that siRNA-mediated knockdown of *POLN* sensitizes human cells to DNA crosslinking agents [11, 12].

**Fig 1:**
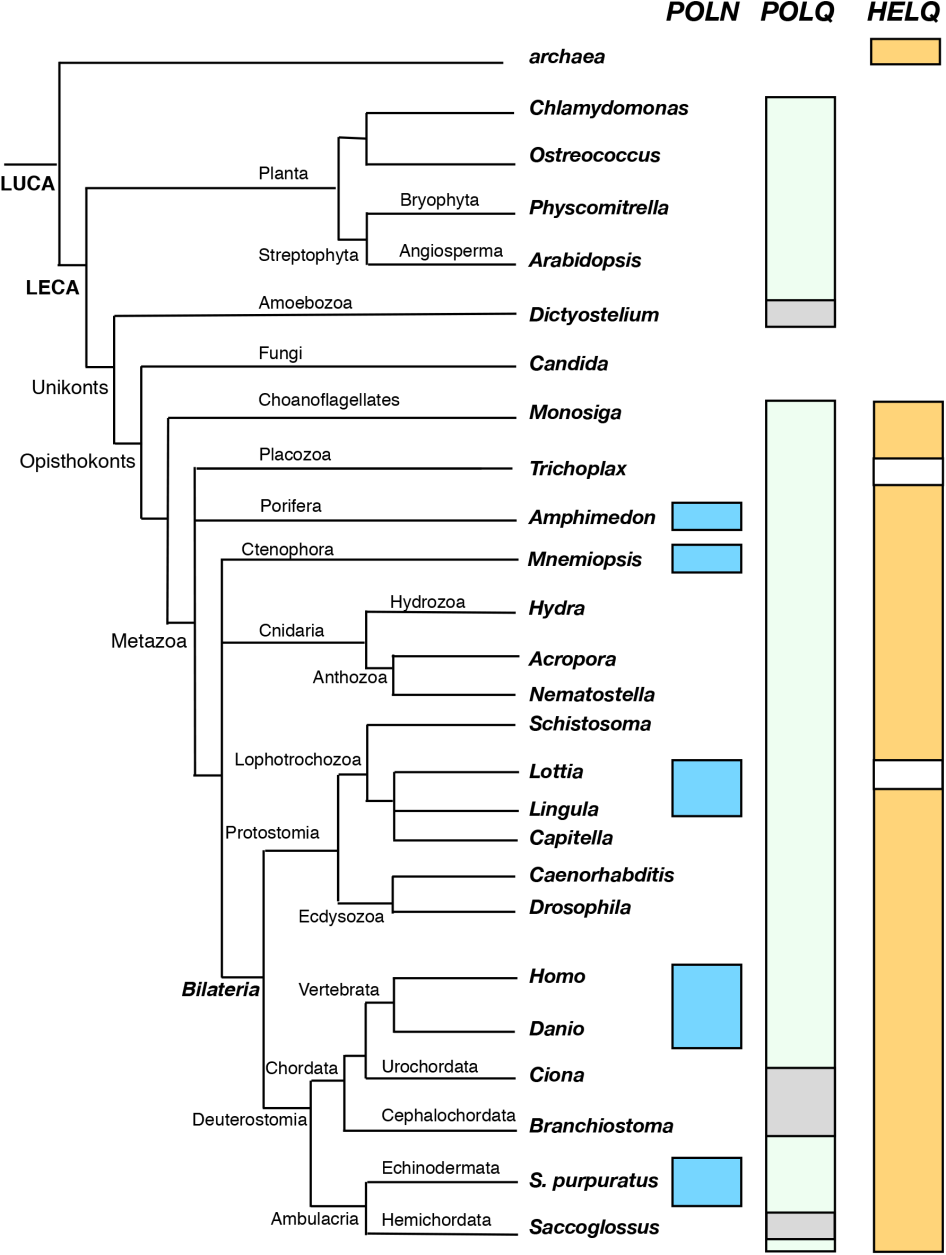
Phylogenetic distribution of *POLN*, *POLQ*, and *HELQ* genes in eukaryotes. Genomic DNA sequence databases for the indicated model organisms, accessed from the NCBI Genome portal, were searched using Blastp for each genus, and then individually verified by alignment. For illustrative purposes, the occurrence of each gene was mapped onto the phylogenetic distribution of the universal stress protein (USP) genes [19]. The presence of *POLN* in the indicated organism is shown by a light blue colored bar, of *POLQ* by a light green bar and *HELQ* by a light orange bar. All identifications were cross-checked and confirmed by accessing Gene Tree view in Ensembl and Ensembl Metazoa (metazoa.ensembl.org). *POLN* genes were distinguished from pol I / polA type homologs frequently present in genomes and plastids of other organisms. Criteria included: 1. The presence of complete sequences for all six conserved polymerase motifs (**S1 Fig**), with key catalytic residues intact. 2. All three sequence inserts present in POLN as described (see S1 Fig in ref [5]). 3. In motif 4 encoding the core of the O-helix, a conserved (K/R)xxY in POLN orthologs, in contrast to (A/T)xxF in pol I orthologs (c.f. Fig 1 in ref [5]). *POLQ* gene distribution indicates presence in the last eukaryotic common ancestor (LECA). In some lineages, shown with grey rectangles, *POLQ* is annotated as split apart into two domains (helicase-like and polymerase like). *POLQ* has been lost in the fungal branch. *HELQ* genes are part of a wider family of helicase genes, with paralogs *ASCC3* and *SNRNP200*. The ancestor of this family may have arisen by lateral gene transfer from archaea, as similar *Hel308* genes are present in both crenarchaea and euryarchaea. *HELQ*, shown here, itself arose in a common metazoan ancestor. *HELQ* genes are absent in several lineages (white rectangles). The relative sister group positions of the Ctenophora and the Porifera with respect to Bilateria are under discussion [20] and are shown illustratively here.

Mammalian genomes encode three Mus308 homologs: *POLN*, *POLQ*, and DNA helicase *HELQ* [3, 13, 14]. Pol θ possesses both a helicase-like and a DNA polymerase domain while pol ν has only a DNA polymerase domain and HELQ has only a helicase domain. Our phylogenetic analysis of the distribution of these three genes is shown in **Fig 1**. *POLQ* genes are found throughout most of the eukaryotic lineage, in both animals and in plants, and are inferred to be present in the last eukaryotic common ancestor, 1500 million years ago. Even the unicellular green algae *Ostreococcus* (the smallest free-living eukaryote) contains *POLQ*. *HELQ* is found in most eukaryotic branches, but not in plants. *HELQ* orthologs are also present in both euryarchaea and crenarchaea [15]. In contrast, *POLN* genes are restricted to a much more limited range of genomes, in some metazoan groups. *POLN* appeared during genomic evolution much more recently than *POLQ*. The *POLN* gene arose, either by gene duplication or horizontal transfer, in the last common ancestor of the metazoa, around 600 million years ago. *POLN* genes are distinguished by characteristic sequence insertions in the polymerase domain (**S1 Fig**), with the location of insert 3 distinct from that of *POLQ* [5].

Human pol ν (900 amino acid residues) has a strong strand-displacement activity and low fidelity, with exceptionally efficient insertion of T opposite template G in the steady state [4, 16, 17]. Purified pol ν can bypass the major groove DNA lesion 5*S*-thymine glycol (Tg) by inserting A, and bypasses major groove DNA-peptide and some DNA-DNA crosslinks [4, 18], but does not bypass other DNA modifications including an abasic (AP) site, a cisplatin-induced intrastrand d[GpG] crosslink, a cyclobutane pyrimidine dimer, a 6–4 photoproduct, or minor groove DNA-peptide or DNA-DNA crosslinks [4, 18]. A unique cavity in the polymerase domain allows pol ν to generate and accommodate a looped-out primer strand [6]. However, it is currently unknown how these unique biochemical properties are implemented *in vivo*.

To help illuminate pol ν function, we examined *POLN* gene expression patterns in mice, and phenotypes of mice and cells where *POLN* was disrupted. The results suggest a specific function in germ cells, but do not support a role for pol ν in tolerance of DNA crosslinks.

## Results

### *Poln* knockout mice have normal development and lifespan

**Fig 2:**
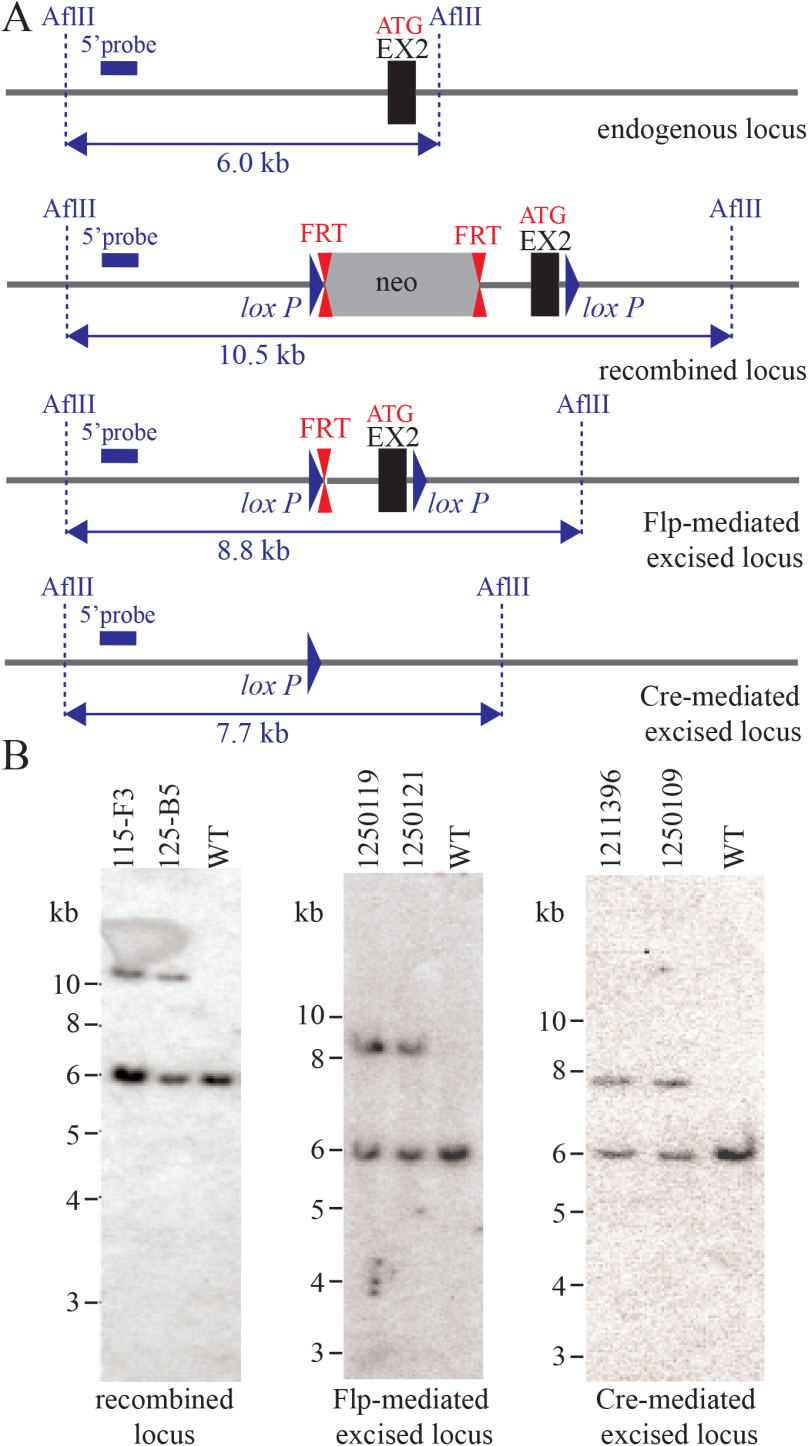
Cre-LoxP mediated disruption of *Poln*. (A) Diagram of the targeted mouse *Poln* allele, with the wild-type (WT) locus shown at the top. The second exon containing the ATG encoding the first methionine (and four other in-frame methionines) is indicated as a black box. Locations of selected restriction sites and the 5’ probe for Southern blotting are shown. In the recombined locus, FRT sites are represented by double red triangles and loxP sites by blue triangles. First, the neomycin positive selection cassette (neo) was excised by breeding with C57BL/6J Flp deleter mice. A subsequent cross with Cre-expressing mice led to excision of the *Poln* exon 2 and surrounding intron sequence. Heterozygous *Poln* knockout mice (*Poln*^+/ΔEx2^) were then used for breeding. (B) Example of Southern blot analysis of (left) the recombined locus (neo+), (middle) the Flp-mediated excised locus and (right) the constitutive *Poln* excised locus. Genomic DNA of the tested animals was compared with C57BL/6J wild-type genomic DNA (WT). AflII-digested DNA was blotted on a nylon membrane and hybridized with the 5’ probe. Restriction fragment sizes were: recombined neo+ locus 10.5 kb, Flp-mediated excised locus 8.8 kb, constitutive *Poln* excised locus 7.7 kb and the wild-type locus 6.0 kb. Genomic DNA was further analyzed extensively and confirmed by specific PCR assays and complete DNA sequencing as described in the Materials and Methods.

**Fig 3:**
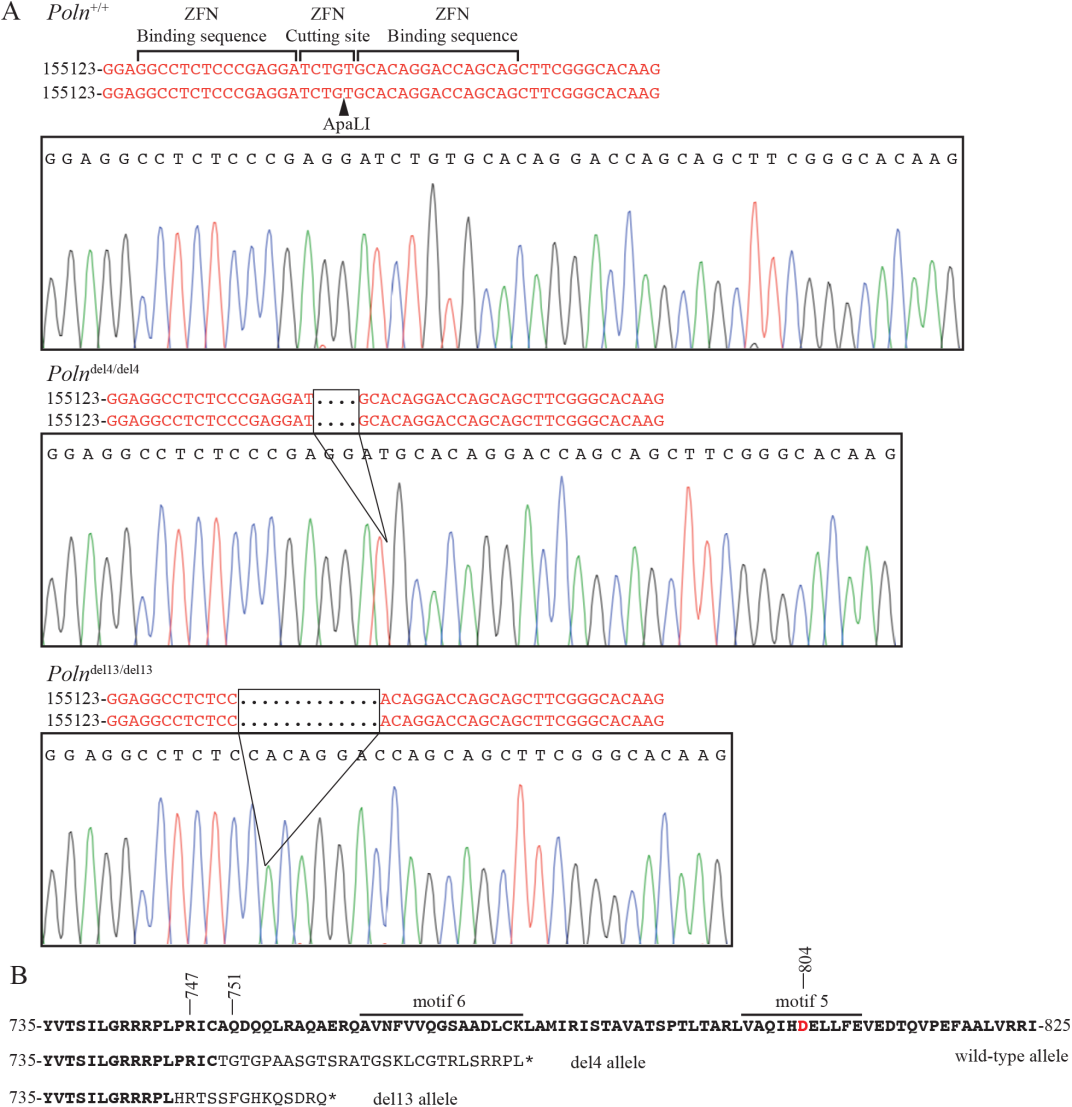
Zinc-finger nuclease (ZFN) targeted disruption of the *Poln* gene. (A) Sequence chromatograms of PCR products for FVB/NCrl wild-type mouse, 4-nt deletion and 13-nt deletion showing the targeted deletions. Nucleobases G, C, A, T are color-coded black, blue, green, and red, respectively. The *Poln*-specific ZFN-binding sites and cutting site are shown on the wild-type allele. An arrowhead indicates the ApaLI restriction enzyme cleavage site used for genotyping. (B). The 4-nt deletion changes the open reading frame after amino acid 751, with a stop codon arising after 778 amino acids (the wild-type allele encodes 866 total amino acids). The 13-nt deletion changes the open reading frame after amino acid 747, with a stop codon arising after 760 amino acids. DNA polymerase motifs and the residue Asp-804 necessary for coordinating bivalent metal ions to interact with an incoming nucleotide are shown in the wild-type sequence. Bold type indicates unaltered sequence.

To explore possible biological functions of pol ν, we established two different *Poln* knockout mouse models. In one model, the second exon was deleted in mice of C57BL/6J;129Sv background (**Fig 2 and S2 Fig**). This exon encodes the ATG initiation codon and the first 46 amino acids, including an N-terminal protein domain that is highly conserved in vertebrates [21]. We avoided targeting the first exon, because *POLN* shares this exon with *HAUS3*, an essential gene encoded within the first intron of *POLN* [21]. In another model, a zinc finger nuclease (ZFN)-mediated 4 or 13 bp frameshift was introduced in the DNA polymerase domain of pol ν (FVB/NCrl background) (**Fig 3A and S3 Fig**). ZFN-mediated *Poln* mutant mice are missing critical parts of the DNA polymerase domain including the highly conserved motifs 5 and 6 (**Fig 3B**). A universally conserved Asp residue in motif 5 of A-family DNA polymerases (D804 in pol ν) that coordinates bivalent metal ions for interaction with an incoming nucleotide, is lost [22]. In all of the engineered mice studied here, a long noncoding RNA gene that overlaps with *Poln* exons 3–6 remains intact.

Both homozygous *Poln*-deficient mouse models were viable and fertile. Comparing them to their wild-type littermates, no reproductive or developmental differences were observed. Heterozygous *Poln* Ex2 mutant mouse crosses yielded pups with normal litter sizes and within expected proportions of genotypes and genders (**Table 1**). Homozygous *Poln* knockout mouse crosses for the ΔEx2, del4 and del13 constructs all yielded normal litter sizes and with normal sized pups at birth. A phenotype analysis, which included a complete gross necropsy, hematology and serum chemistry, full histopathology on all tissues, and survey radiographs was completed with adult ZFN-mediated *Poln* knockout mice. A comparison between wild-type mice and *Poln*-deficient mice did not reveal any obvious differences in size, body weight, organ development (**Table 2**), or skeletal appearance. Additionally, no significant differences between genotypes were observed in hematology or serum chemistry analysis (**Table 2**).

**Table 1:** Percentages of genotypes produced from heterozygous *Poln* crosses.

Genotype and gender of mice produced by *Poln*^+/ΔEx2^ breeders					
	Male	Female	Total	Expected (%)	Observed (%)
***Poln***^**+/ΔEx2**^	105	85	190	50	56
***Poln***^**ΔEx2/ΔEx2**^	35	41	76	25	22
***Poln***^**+/+**^	41	32	73	25	22
**Total**	181	158			
**Expected (%)**	50	50			
**Observed (%)**	53	47			

**Table 2:** Phenotype analysis.

	Female		Male	
Serum chemistry	WT	KO	WT	KO
Body weight (g)	20.25 ± 2.13	23.98 ± 1.39	26.41 ± 1.20	27.74 ± 0.87
Spleen %	0.43 ± 0.03	0.45 ± 0.04	0.39 ± 0.07	0.38 ± 0.02
Liver %	5.08 ± 0.29	5.57 ± 0.16	5.73 ± 0.46	5.40 ± 0.14
Kidney %	1.29 ± 0.08	1.31 ± 0.09	1.89 ± 0.21	1.68 ± 0.18
Adrenals %	0.061 ± 0.001	0.061 ± 0.008	0.023 ± 0.004	0.036 ± 0.005
Thymus %	0.23 ± 0.06	0.23 ± 0.04	0.17 ± 0.04	0.15 ± 0.02
Heart %	0.53 ± 0.05	0.49 ± 0.08	0.50 ± 0.05	0.49 ± 0.03
Brain %	1.62 ± 0.08	1.48 ± 0.15	1.39 ± 0.11	1.26 ± 0.02
Testes %			0.69 ± 0.06	0.69 ± 0.04
Ovaries %	0.095 ± 0.022	0.099 ± 0.018		
**Hematology**				
RBC (x 10^6^/μL)	9 ± 0.3	8.6 ± 1.1	8.8 ± 0.3	9 ± 0.3
Hemoglobin (g/dL)	14.6 ± 0.2	14.9 ± 0.6	13.8 ± 0.6	13.7 ± 0.5
Hematocrit (%)	45 ± 1.2	42 ± 1.3	41 ± 1.2	42 ± 1.7
MCV (fL)	49.4 ± 0.5	48.7 ± 4.7	46.4 ± 0.6	46.8 ± 0.2
MCH (pg)	16.2 ± 0.4	17.4 ± 2.4	15.7 ± 1.0	15.3 ± 0.4
MCHC (g/dL)	32.8 ± 0.5	35.6 ± 1.8	34 ± 2.2	32.7 ± 1.0
RDW (%)	12.7 ± 0.3	12.5 ± 0.1	12.6 ± 0.8	12.3 ± 0.4
Platelet count (x 10^3^/μL)	1471 ± 224	1610 ± 297	1614 ± 170	1825 ± 109
MPV (fL)	8.8 ± 0.9	7.7 ± 1.6	6.8 ± 0.7	6.4 ± 0.2
WBC (x 10^3^/μL)	6.2 ± 1.0	7.9 ± 1.7	6.4 ± 1.5	6.8 ± 1.4
Seg Neutrophils (%)	7.3 ± 2.5	14.3 ± 5.8	15 ± 6.6	16 ± 2.7
Band Neutrophils (%)	0	0	0	0
Lymphocytes (%)	89 ± 2.7	81 ± 4.9	80 ± 8.6	80 ± 2.5
Monocytes (%)	1.7 ± 1.2	3.3 ± 0.6	2.3 ± 3.2	2.3 ± 0.6
Eosinophils (%)	2 ± 3.5	1.7 ± 1.5	2 ± 1.0	2 ± 1.7
Basophils (%)	0	0	0.3 ± 0.6	0
Metamyelocytes %	0	0	0	0
**Serum chemistry**				
ALB (g/dL)	4.1 ± 0.1	4 ± 0.1	3.4 ± 0.2	3.4 ± 0.2
ALP (u/L)	126 ± 9.1	115 ± 11.5	88 ± 15.3	83 ± 19.4
ALT (u/L)	43 ± 2.1	60 ± 24.1	56 ± 4.9	56 ± 1.5
AMY (u/L)	1252 ± 132	1259 ± 41	1588 ± 432	1410 ± 137
TBIL (mg/dL)	0.4 ± 0.1	0.3 ± 0	0.4 ± 0.1	0.3 ± 0
BUN (mg/dL)	19 ± 4.0	17 ± 1.5	24 ± 0.6	23 ± 4.7
CA (mg/dL)	10.6 ± 0.5	11 ± 0.4	10.4 ± 0.2	10.4 ± 0.5
PHOS (mg/dL)	15.6 ± 7.6	10.1 ± 1.2	9.3 ± 1.9	9 ± 1.2
CRE (mg/dL)	<0.2	<0.2	<0.2	<0.2
GLU (mg/dL)	228 ± 7.8	243 ± 27.2	245 ± 15.6	275 ± 44.4
NA (mmol/L)	156 ± 2.1	154 ± 3.1	159 ± 2.3	157 ± 1.2
K (mmol/L)	>8.5	8.5	>8.5	8.4
TP (g/dL)	5.6 ± 0.1	5.9 ± 0.1	5.2 ± 0.2	5.3 ± 0.1
GLOB (g/dL)	1.4 ± 0.2	1.8 ± 0.1	1.8 ± 0.2	1.9 ± 0.1
A/G	2.9 ± 0.3	2.2 ± 0.1	1.8 ± 0.2	1.8 ± 0.2

Mean body weights, organ weights, hematology and serum chemistry results for adult 10-week-old *Poln*^*del4/del4*^ (KO) and *Poln*^+/+^ (WT) mice of both sexes. RBC: red blood cell count, MCV: mean corpuscular volume, MCH: mean corpuscular hemoglobin, MCHC: Mean corpuscular hemoglobin concentration, RDW: red cell distribution width, MPV: mean platelet volume, WBC: white blood cell count, ALB: albumin, ALP: alkaline phosphatase, ALT: alanine aminotransferase, AMY: amylase, TBIL: total bilirubin, BUN: blood urea nitrogen, CA: calcium, PHOS: phosphorus, CRE: creatinine, GLU: glucose, NA: sodium, K: potassium, TP: total protein, GLOB: globulin, A/G: albumin/globulin ratio. Mean calculated from 3 mice of each gender and genotype (± SD). Organ weights recorded as percent total body weight.

**Fig 4:**
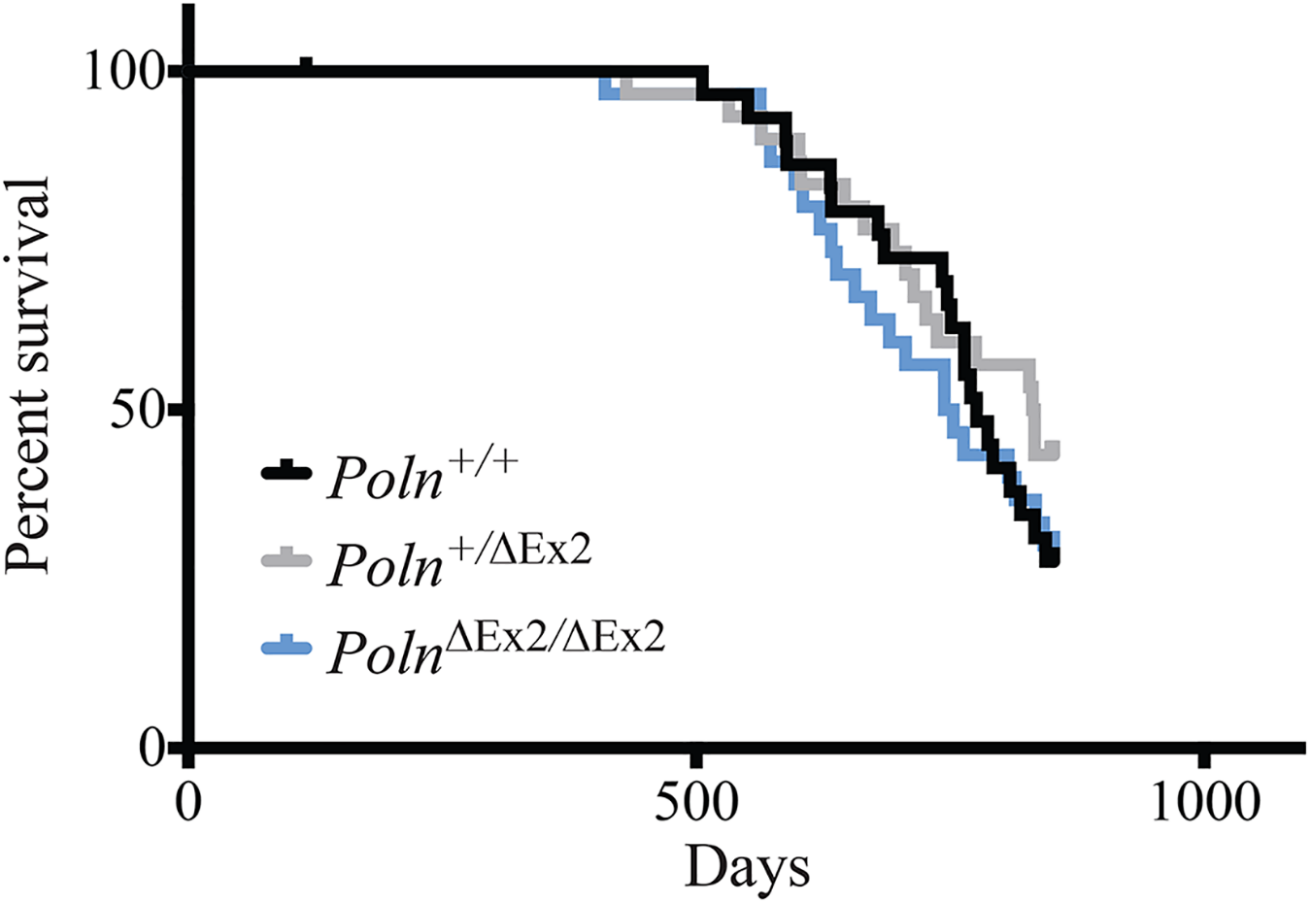
*Poln* does not affect overall survival. Kaplan-Meier plot of morbidity-free survival for *Poln*^+/+^, *Poln*^+/ΔEx2^, and *Poln*^ΔEx2/ΔEx2^ mice (n = 30 for each genotype). The study was terminated at 850 days.

*Poln*^+/+^, *Poln*^+/ΔEx2^, and *Poln*^ΔEx2/ΔEx2^ mice were monitored for over 2 years to assess survival, general health, and tumor incidence. Loss of functional *Poln* did not affect overall survival compared to wild-type and heterozygous littermate controls (**Fig 4**). There were no significant differences in tumor prevalence, multiplicity or distribution of tumor types (**Tables 3 and 4**). Non-neoplastic lesions were of types and incidence commonly found in aging laboratory mice, and there appeared to be no pattern of susceptibility related to genotype (**Table 5**) or sex. All mice had a variety of age-related lesions that were considered incidental and not included in non-neoplastic diagnoses. These included atrophies of testicles, ovaries, uterus, and thymus, mild multifocal lymphoid infiltrates in various organs, and mild glomerulopathy. Similarly, no obvious alterations in lifespan, general health or cause of death were noted in *Poln* del4 and del13 mice.

**Table 3:** Incidence of neoplastic findings in *Poln*^ΔEx2/ΔEx2^ (KO) *Poln*^+/ΔEx2^ (HET), and *Poln*^+/+^ (WT) mice at end of life.

Genotype	Number of mice analyzed	HS	TCRBCL	BA Adenoma	BA Carcinoma	Hepatocellular Adenoma	Chromophobe Adenoma	Other Tumor
KO	21	4	10	1	1	2	0	1
		(19)	(48)	(5)	(5)	(10)	(0)	(5)
HET	17	1	5	3	1	0	1	2
		(6)	(29)	(18)	(6)	(0)	(6)	(12)
WT	19	5	6	2	1	1	2	1
		(26)	(32)	(11)	(5)	(5)	(11)	(5)

Table shows number of mice with indicated tumor type evaluated at end of life (moribund status). There were no significant differences in genotype or tumor type comparisons (Fisher’s exact test). Percentages are shown in parentheses. HS = histiocytic sarcoma; TCRBCL = T-cell-rich B cell lymphoma; BA = bronchioloalveolar.

**Table 4:** Tumor multiplicity of *Poln*^ΔEx2/ΔEx2^, *Poln*^+/ΔEx2^, and *Poln*^+/+^ mice at end of life.

Genotype	Number of mice analyzed	Number of mice with tumors	Total tumors	# Tumors per tumor-bearing animal
KO	21	15	19	1.3
		(71)		
HET	17	10	16	1.6
		(59)		
WT	19	15	18	1.2
		(79)		

The table shows total number of mice analyzed, number of mice with tumors, total number of tumors observed by genotype, percentage of animals analyzed found to have tumors (shown in parentheses) and frequency of tumor occurrence per tumor-bearing animal. There was no significant difference between genotypes in tumor multiplicity (pairwise comparison using Wilcoxon rank sum test).

**Table 5:** Incidence of non-neoplastic findings in *Poln*^ΔEx2/ΔEx2^, *Poln*^+/ΔEx2^, and *Poln*^+/+^ mice at end of life.

Genotype	Number of mice analyzed	Pneumonia	Pancreatic Acinar Atrophy	Urinary obstruction	Heart disease	Dermatitis	Renal amyloidosis	Other
KO	21	4	2	4	5	4	2	3
		(19)	(10)	(19)	(24)	(19)	(10)	(14)
HET	17	8	4	2	3	0	1	7
		(47)	(24)	(12)	(18)	(0)	(6)	(41)
WT	19	7	0	3	4	4	1	5
		(37)	(0)	(16)	(21)	(21)	(5)	(26)

Table shows number of mice with indicated condition evaluated at end of life (moribund status). A comparison between genotypes and conditions by two independent proportions test revealed no significant differences between groups. Percentages are shown in parentheses.

### *Poln* has high relative expression in spermatocytes, but is not essential for spermatogenesis and meiotic recombination

**Fig 5:**
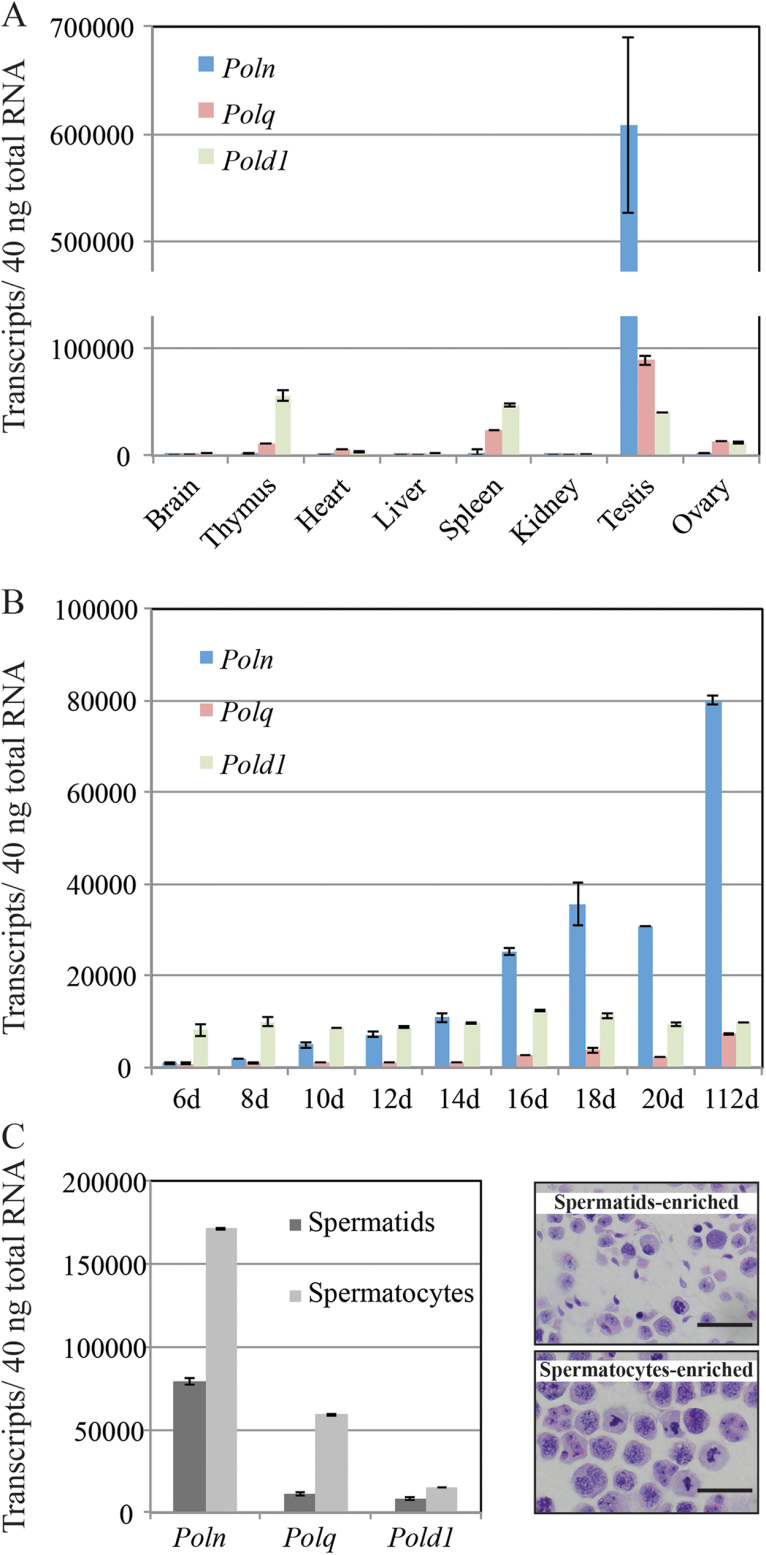
Preferential expression of *Poln* in testis. (A) Amount of *Poln*, *Polq*, and *Pold1* mRNA in organs from 40 day-old FVB/NCrl mice. Brain, bone marrow, ovaries, spleen, kidney, liver, and lung were isolated from a female. Thymus, heart, skeletal muscle, and testis were isolated from a male. (B) Amount of *Poln*, *Polq*, and *Pold1* mRNA during testis development of FVB/NCrl mice. (C) Expression in separated testicular cells or fractions enriched in spermatids and pachytene spermatocytes from FVB/NCrl mice. The y-axis indicates the absolute quantity of transcripts for *Poln*, *Polq*, and *Pold1* per 40 ng of total RNA isolated from samples. In C (right panel), each fraction was visualized in cell smears stained with periodic acid Schiff-hematoxylin. Scale bar, 20 μm.

We previously reported that the *POLN* transcript is most prominent in adult testis from the human, mouse, and zebrafish [3, 21]. To quantify absolute transcript levels, we compared expression of *Poln*, *Polq*, and the replicative polymerase *Pold1* in different mouse tissues. *Poln* expression was highest in testis by an order of magnitude, much higher than *Polq* and *Pold1* in this organ. However, *Poln* transcript was almost undetectable in other tissues, in contrast to *Polq* and *Pold1* (**Fig 5A**). To investigate when *Poln* starts expressing during testis development, we isolated testes from young male mice of various ages. *Poln* expression gradually increased and was higher than *Polq* and *Pold1* after 16-days, when the population of spermatocytes in the pachytene stage increases [23]. In contrast, the amounts of mRNA of *Polq* and *Pold1* were not markedly increased during testis development (**Fig 5B**). Fractions enriched in spermatids and pachytene spermatocytes were prepared by centrifugal elutriation, and *Poln* expression was higher in the pachytene-enriched fraction (**Fig 5C**). These data suggested a possible meiosis-related function of pol ν in testis.

**Fig 6:**
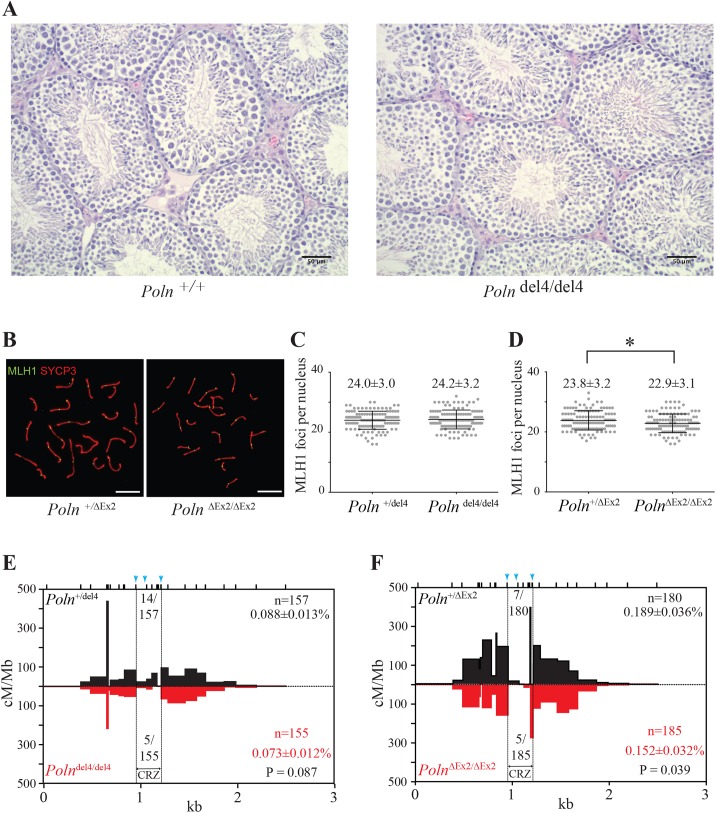
Testis morphology and meiotic recombination in *Poln* knockout mice. (A) Testis sections were made and H & E stained from *Poln*^+/+^ and *Poln*^del4/del4^ FVB/NCrl adult male mice (10-weeks old). These mice were from the phenotype analysis (Table 2). Scale bar: 50 μm. (B) Double-immunolabelling of SYCP3 (red) and MLH1 (green) in *Poln*^+/ΔEx2^ or *Poln*^ΔEx2/ΔEx2^ spread spermatocytes. Scale bar: 10 μm. (C and D) Number of MLH1 foci per nucleus of *Poln*^+/del4^ and *Poln*^del4/del4^ FVB/NCrl x DBA/2J mice (C) or *Poln*^+/ΔEx2^ and *Poln*^ΔEx2/ΔEx2^ in C57BL/6J x DBA/2J mice (D), *p<0.05 by Mann-Whitney *U* test. (E and F) Total crossover breakpoints are mapped in the bar graphs, (E): *Poln*^+/ del4^ and *Poln*^del4/del4^ in FVB/NCrl x DBA/2J F1 hybrid sperm, (F): *Poln*^+/ΔEx2^ and *Poln*^ΔEx2/ΔEx2^ in C57BL/6J x DBA/2J F1 hybrid sperm. Numbers of crossovers examined, Poisson-adjusted frequencies (± SD), and p-values (Fisher’s exact test) are indicated. Ticks (black) represent positions of the tested polymorphisms. Arrows (blue), insertion/deletion polymorphisms.

Testis morphology was normal in *Poln* knockout mice (**Fig 6A**), and testes size measurements, histology and breeding experiments described above did not suggest a marked perturbation of spermato- and spermiogenesis. To test for an overt role of pol ν in meiotic recombination, chromosome spreads were stained with SYCP3 (a component of the synaptonemal complex) and MLH1 (a crossover marker) to assess progression through meiotic prophase I (**Fig 6B**). MLH1 foci numbers per nucleus were not statistically different between pol ν proficient and *Poln*^del4/del4^ mice (**Fig 6C)**. *Poln*^ΔEx2/ΔEx2^ spermatocytes had one less MLH1 focus per nucleus than controls (p = 0.03) (**Fig 6D**). The results suggest that while pol ν is not essential for crossing over or meiosis, it may be required for efficient crossing over at specific loci, at least in some genetic backgrounds (see legend to S4 Fig)

### Possible function of pol ν at a meiotic recombination hotspot

We examined the potential role of pol ν in meiotic homologous recombination-associated DNA synthesis. Crossovers, arising by recombination at one meiotic hotspot *A3* [24, 25], were isolated from sperm of *Poln*^ΔEx2/ΔEx2^ C57BL/6J x DBA/2J and *Poln*^del4/del4^ FVB/NCrl x DBA/2J F1 hybrid mice, and control animals. The crossover frequency at *A3* in FVB/NCrl x DBA/2J is half that of C57BL/6J x DBA/2J (**Fig 6E and 6F**). The *A3* locus in FVB/NCrl lacks a high affinity PRDM9 binding site (**S4 Fig**) and, therefore it likely does not receive meiotic DSBs to initiate recombination [26]. As such, the FVB/NCrl x DBA/2J F1 hybrid receives meiotic DSBs on only the DBA/2J allele, rather than at both the C57BL/6J and DBA/2J alleles as in the C57BL/6J x DBA/2J F1 hybrid. Consistent with this model, the *A3* locus shows reciprocal crossover asymmetry, an altered distribution of crossovers depending upon their orientation, which is a hallmark of meiotic hotspots with biased DSB formation on only one of the two parental alleles (**S4 Fig**).

A total of 155, 157, 185, 180 crossovers from *Poln*^del4/del4^, *Poln*^+/del4^, *Poln*^ΔEx2/ΔEx2^, and *Poln*^+/ΔEx2^ males were isolated and mapped. Consistent with reduced MLH1 foci, the Poisson corrected crossover frequency in male *Poln*^ΔEx2/ΔEx2^ mice was 80% of that in heterozygous littermates (p = 0.04) (**Fig 6F**). The crossover frequency in *Poln*^del4/del4^ mutants was not significantly reduced compared to controls (p = 0.09, **Fig 6E**), but combining both datasets showed a statistically significant (p = 0.01) reduction in *Poln* homozygous mutants to about 80% of the control frequency (**S5 Fig**).

We further examined the so-called ‘crossover refractory zone’ (CRZ) at the *A3* hotspot in *Poln* mutant mice. Crossover formation in this 258 bp region of the *A3* hotspot is strongly inhibited, even though DSBs are formed there abundantly [24, 27]. The region contains secondary structure-forming sequence that may inhibit heteroduplex formation [24]. The combined *Poln* mutant data (**S5 Fig**) showed a statistically significant (p = 0.04) 50% reduction in crossover frequency at this region (10 of 340 crossovers in knockouts, 21 out of 337 crossovers in controls).

### *Poln* deficiency does not perturb transcription in testis

**Fig 7:**
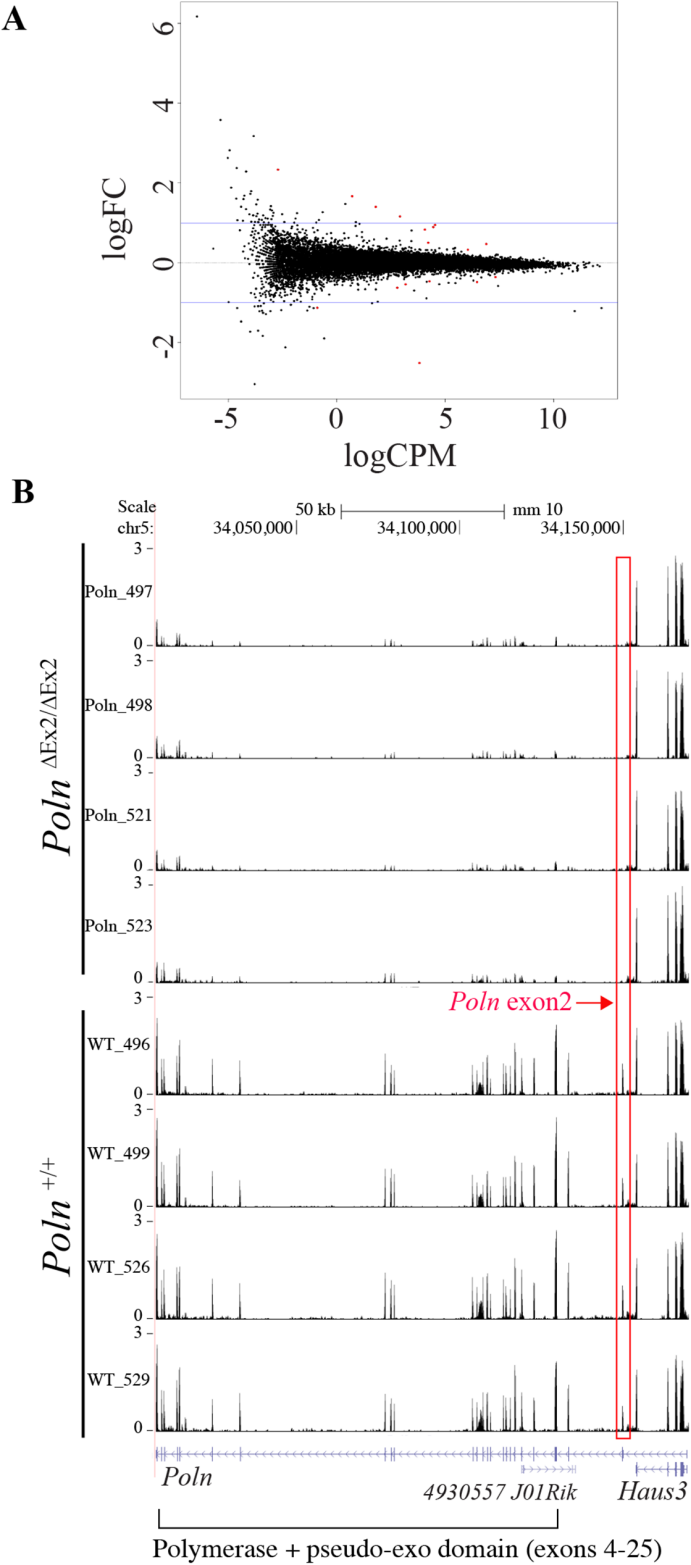
Transcriptional profiling of *Poln* deficient mouse testes by RNAseq. (**A**) X-axis: average transcription levels (log2) in CPM (counts per million library reads). Y-axis: Fold change (FC, log2) in transcription, comparing *Poln*^+/+^ and *Poln*^ΔEx2/ΔEx2^ testes. Positive values indicate genes with higher transcription in the absence of pol ν, negative values indicate lower transcription. Black dots indicate genes with no significant differential transcription; red dots indicate differentially expressed genes (p < 0.05). (**B**) Genome browser screenshots of the *Poln* /*Haus3* locus showing RNA-seq data for four wild-type and four *Poln* disrupted mice. The red box highlights exon 2 knocked out in the *Poln* mutant mice. This exon contains the initiation codon and a conserved N-terminal domain [21]. The globular DNA polymerase / pseudo-exonuclease domain [6] is encoded by exons 4–25. A long non-coding RNA gene (4930557 J02Rik) is specified by the strand complementary to *Poln* exons 3–6.

Whole genome transcriptome analysis by RNAseq was performed to determine whether *Poln* disruption influences gene expression in testis from *Poln*^ΔEx2/ΔEx2^ mice. Using rRNA-depleted total RNA, we obtained an average of 65 million reads per sample (range 53 to 73 million) with an average mapping rate of 90% to the reference mouse genome. *Poln* disruption did not perturb overall transcription (**Fig 7A**). Only a few genes had significantly altered expression, at a false discovery rate (FDR) ≤ 0.05. Expression of only four of these genes was increased more than 2-fold (**S1 Table**), comprising two that encode proteins *(Lgi2* and *9330182L06Rik*), and two specifying noncoding RNAs (*BB283400* and *Speer4cos*). Expression of only two genes was reduced more than 2-fold, *Egfem1* and *Poln* itself. Because these genes have no close physical linkage and their functions are unknown, we have not pursued them further at present. As expected, expression of the targeted exon 2 of *Poln* was not detected in *Poln*^ΔEx2/ΔEx2^ mice. All other *Poln* exons showed greatly reduced or absent expression, likely related to the disruption of normal splicing. *Haus3* expression was not influenced by deletion of the targeted *Poln* exon (**Fig 7B**).

### *Poln* deficiency does not influence radiation-induced DNA damage levels in spermatids

**Fig 8:**
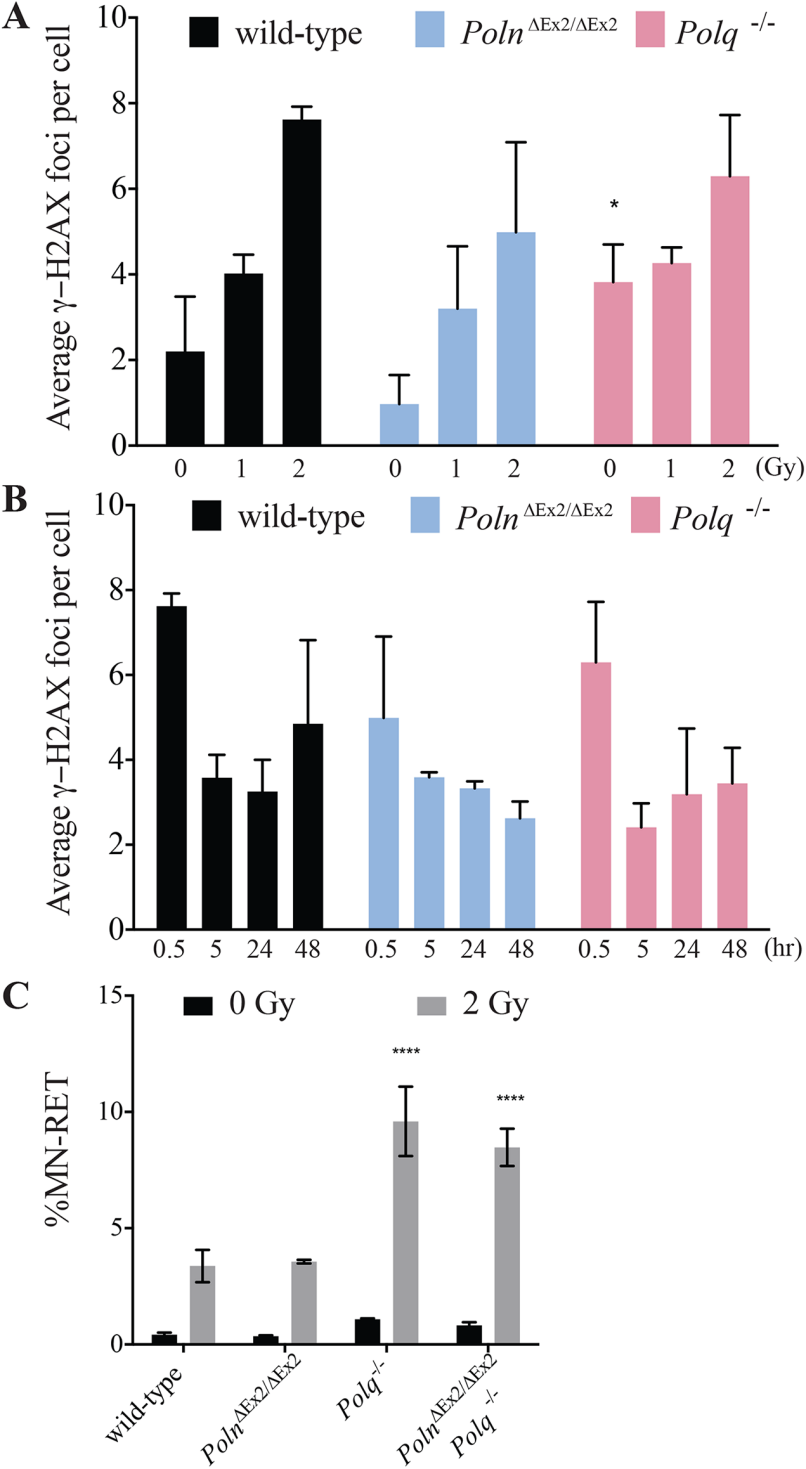
Induction and repair of radiation-induced DNA damage in round spermatids. (A) Dose-dependent induction of radiation-induced γ-H2AX foci. Data are presented as means from three mice ± SE. (B) DNA repair kinetics. At defined time-points after irradiation with 2 Gy, round spermatids were analyzed by counting foci from three mice ± SE; *significant difference (p < 0.01) compared with wild-type control. (C) Spontaneous and radiation-induced micronucleus frequencies were measured in reticulocytes. Data are presented as means from three mice ± SE; **** significant difference (p < 0.0001) compared with wild-type control.

We asked whether pol ν influences levels of ionizing radiation-induced DNA damage in testis. This could be the case if pol ν is directly involved in DSB repair (by analogy with the related pol θ) or if pol ν is necessary for translesion DNA synthesis of radiation-induced lesions such as thymine glycol (Tg). Pol ν is proficient in bypass of Tg, which blocks the progression of replicative DNA polymerases [4]. A dose dependent increase in γ-H2AX foci (a surrogate for DNA damage including DSB) was observed in wild-type, *Poln*^ΔEx2/ΔEx2^, and *Polq*^-/-^ mice (**Fig 8A**). The number of γ-H2AX foci in the absence of irradiation was higher in *Polq*^-/-^ mice than in wild-type or *Poln*^ΔEx2/ΔEx2^ mice. This is consistent with the higher basal level of γ-H2AX foci in pol θ-suppressed human cells [28]. Following irradiation of mice with 1 or 2 Gy, the average γ-H2AX foci numbers in round spermatids of testis sections was measured [29]. After 5 hr, the number of foci was reduced to a similar level in all mice (**Fig 8B**), irrespective of *Poln* and *Polq* status. As a control experiment, blood was taken from killed mice after irradiation and micronuclei were measured in reticulocytes. Frequencies of micronuclei were increased in *Polq*^-/-^ mice after IR, as previously reported [10, 30, 31]. *Poln* status did not influence the level of micronuclei in reticulocytes (**Fig 8C**). We also examined *Poln*^ΔEx2/ΔEx2^ *Polq*^-/-^ double knockout mice and found that *Poln* deletion did not further increase the frequency of micronuclei above that in *Polq*^-/-^ reticulocytes (**Fig 8C**).

### *Poln* disruption does not influence mouse immunoglobulin class-switching

**Fig 9:**
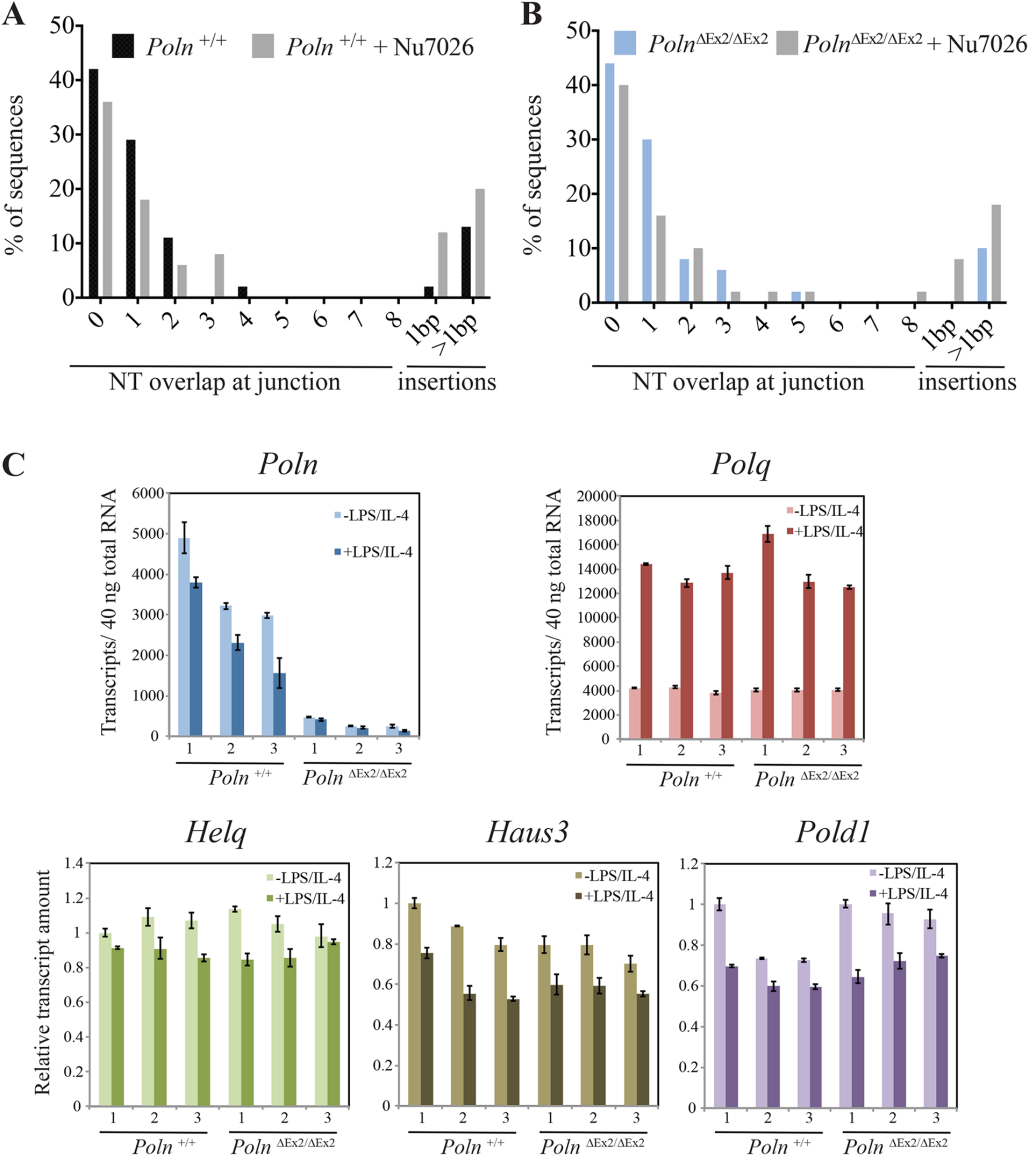
*Poln* does not influence insertions at CSR junctions. Genomic DNA was isolated from naïve splenic B cells of wild-type (A) and *Poln*^ΔEx2/ΔEx2^ (B) mice stimulated to undergo CSR to IgG1 and either mock treated or NU7026 treated. Sμ-Sγ1 junctions were amplified by PCR and 100 Sμ-Sγ1 junctions from each group were sequenced and analyzed for overlaps and insertions at breakpoints. (C) Real-time PCR analysis during CSR. The y-axis indicates transcripts per 40 ng of total RNA isolated from B-cells isolated from wild-type and *Poln*-mutant mice. B-cells were either unstimulated or activated by lipopolysaccharide (LPS) and IL-4 to stimulate CSR. *Poln* and *Polq* transcript numbers were detected by absolute quantification. *Helq*, *Haus3*, and *Pold1* transcripts were determined by relative quantification.

Because pol θ can participate in end-joining of DNA breaks produced during immunoglobulin class-switch recombination (CSR) [8], we tested whether pol ν is also involved in CSR. Naïve B cells isolated from the spleens of wild-type and *Poln*^ΔEx2/ΔEx2^ mice were stimulated for IgM to IgG class switching, and then the fraction of IgG1-positive B cells was measured by flow cytometry. Parallel B-cell cultures were incubated with NU7026, an inhibitor of DNA-PKcs that increases the proportion of CSR with >1 bp insertion at the junction [8, 32]. We found that stimulated B cells from *Poln*-proficient and deficient mice had similar overall frequencies of IgG1 (6.1%). Inhibition of DNA-PKcs increased the frequency of CSR in both genotypes by 2-fold (11.7% in wild-type, 12.5% in *Poln*^ΔEx2/ΔEx2^) **(S2 Table)**, as observed previously with wild-type and *Polq*^-/-^ mice [8]. The Sμ-Sγ1 junction was then sequenced from 100 clones of each group of IgG1-positive B cells. Insertions of >1 bp at Sμ-Sγ1 junctions were pol θ-dependent [8], but pol ν-independent (**Fig 9A and 9B**). We also performed qPCR analysis of transcript expression in splenic B cells for *Polq*, *Poln*, *Helq*, *Haus3*, and *Pold1*. Interestingly, among those genes only *Polq* expression was increased after B-cell activation by lipopolysaccharide and interleukin 4 treatment (**Fig 9C**).

### *Poln* deficient cells have a normal response to DNA damaging agents

**Fig 10:**
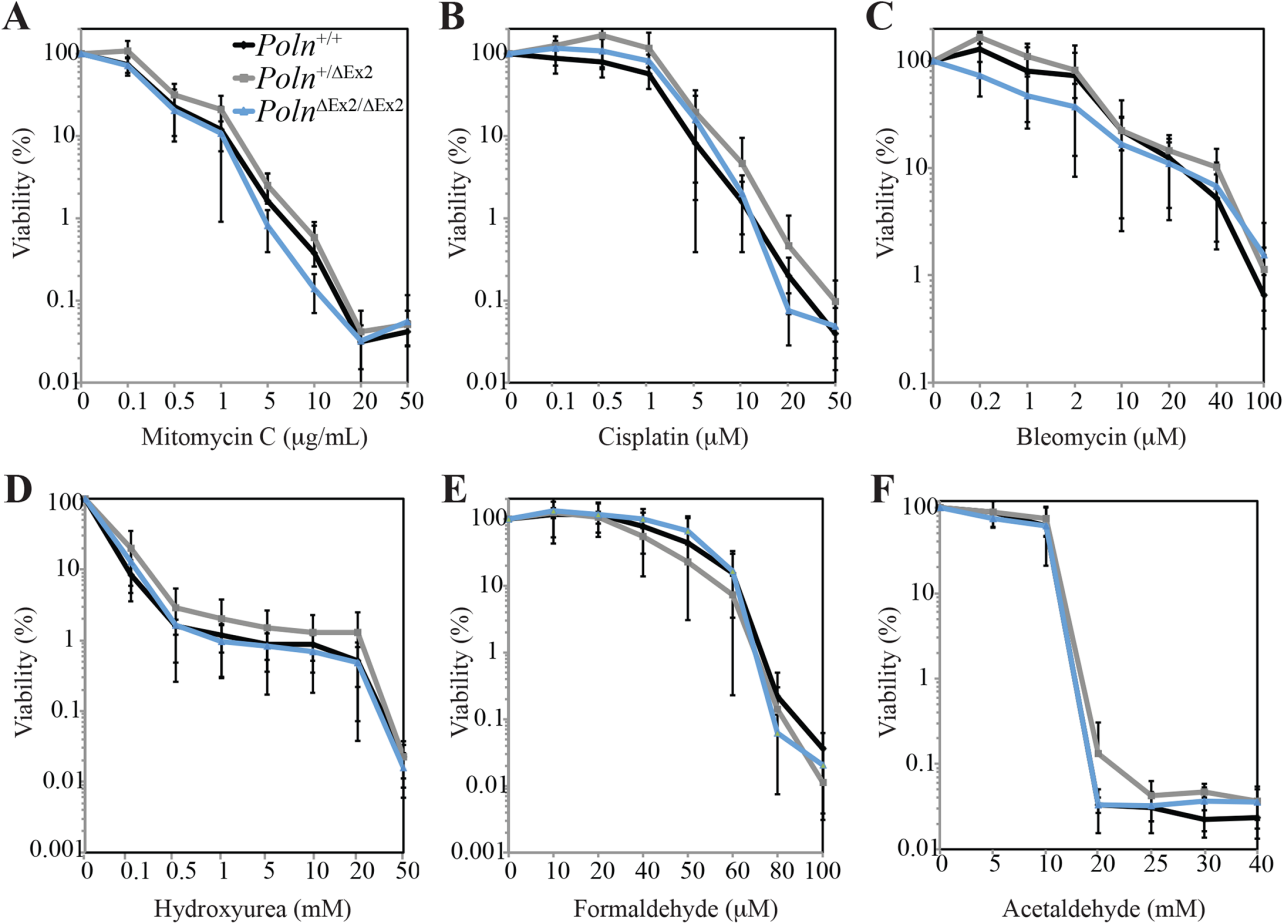
The inactivation of pol ν does not sensitize Tag immortalized mouse embryonic fibroblasts to DNA damaging agents. Cells were exposed to indicated doses of mitomycin C for 48 hr (A), cisplatin for 48 hr (B), bleomycin for 48 hr (C), hydroxyurea for 48 hr (D), formaldehyde for 96 hr (E) and acetaldehyde for 72 hr (F). Viability was determined by measuring ATP content as described in Materials and Methods. *Poln*^+/+^ MEFs (black), *Poln*^+/ΔEx2^ MEFs (grey) and *Poln*^ΔEx2/ΔEx2^ MEFs (blue). The mean is shown for three independently derived MEFs for each genotype, separately plated in triplicate and treated, with SD indicated by error bars.

Cells with inactivation of pol θ or its ortholog HELQ show increased sensitivity to DNA damaging agents, such as bleomycin (pol θ) [8] and mitomycin C or cisplatin (HELQ) [33–35]. To determine whether inactivation of pol ν also sensitizes cells to DNA damage, we examined the relative sensitivity of *Poln*^ΔEx2/ΔEx2^ MEFs to mitomycin C (MMC), cisplatin, bleomycin, hydroxyurea, olaparib, formaldehyde and acetaldehyde. Aldehyde sensitivity was tested because of the observation that purified pol ν can bypass some DNA-peptide crosslinks [18]. However, we detected no hypersensitivity of immortalized MEFs to any of the tested DNA damaging agents (**Fig 10**). We also examined primary *Poln*^del4/del4^ MEFs and found no hypersensitivity to MMC, olaparib, etoposide or 5-fluorouracil (**S6 Fig**). *Rev3L* null MEFs were used as positive controls in the same experiment, and they showed sensitivity to MMC as reported [36]. We generated *Poln*^ΔEx2/ΔEx2^ *Polq*^−/−^ MEFs by crossing *Poln*^ΔEx2/ΔEx2^ and *Polq*^−/−^ mice, isolating double mutant MEFs from the progeny and immortalizing with SV40 Tag. *Polq*^−/−^ MEFs were more sensitive to bleomycin, as reported [8]. *Poln*^ΔEx2/ΔEx2^ MEFs were not more sensitive to bleomycin and *Poln* ablation did not further increase the bleomycin sensitivity of *Polq*^−/−^ MEFs (**S7A Fig**). The cellular sensitivity to MMC was not increased in *Poln* or *Polq* single knockout MEFs or in the double homozygous mutants (**S7B Fig**).

**Fig 11:**
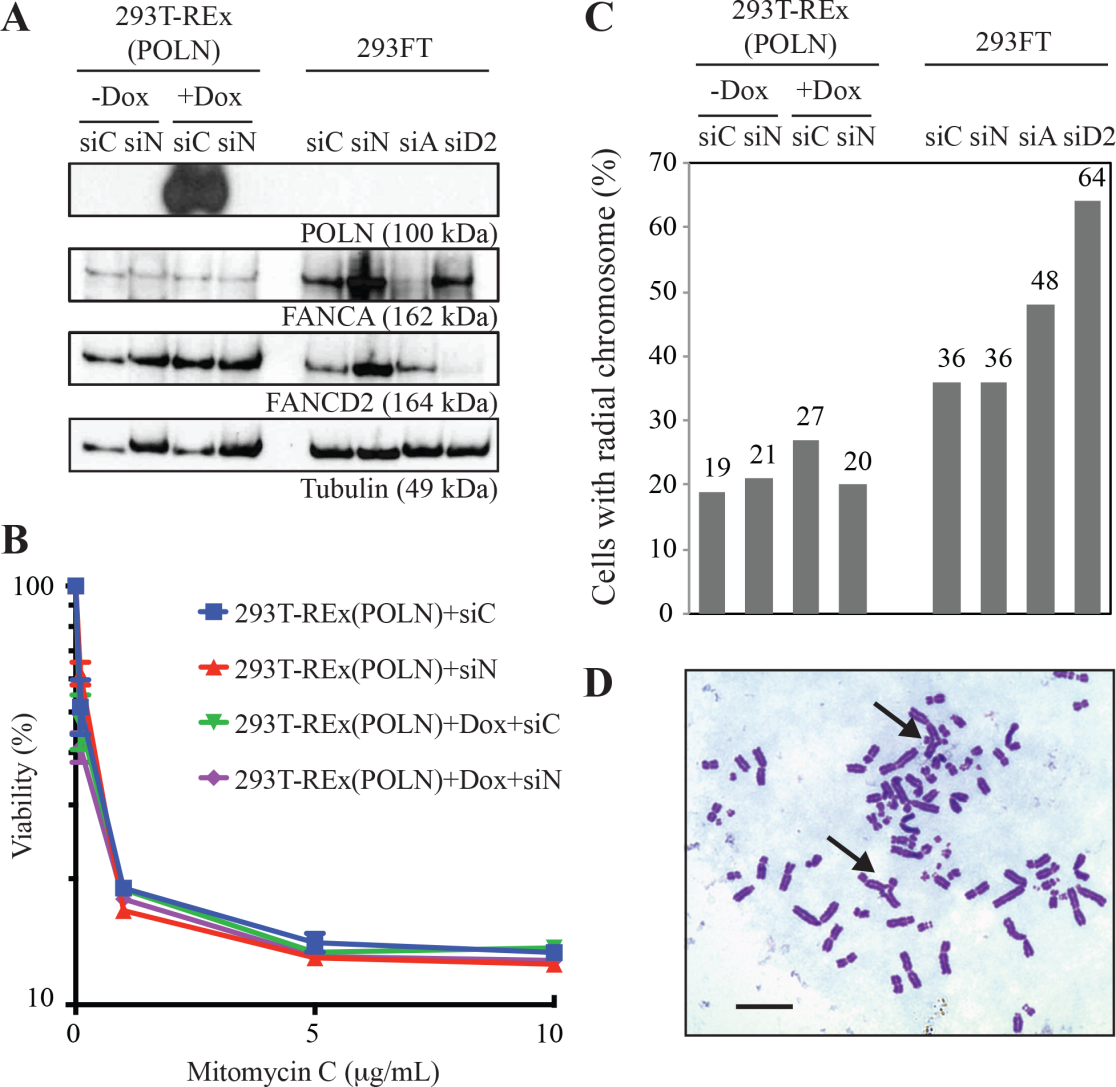
Depletion of pol ν from human cells does not change cellular sensitivity to mitomycin C or alter the frequency of mitomycin C-induced radial chromosomes. (A) Upper panel: immunoblot showing efficacy of siRNA-mediated knockdown of POLN (siN), FANCA (siA), and FANCD2 (siD2) in 293FT cells and 293T-REx doxycycline inducible POLN cells. siC served as a negative control and Tubulin as loading control. Polyclonal anti-pol ν antibody (PA434) recognized overexpressed pol ν [3] but not endogenous pol ν. (B) Cells were exposed to the indicated doses of mitomycin C for 48 hr. Viability was determined by measuring ATP content as described in Materials and Methods. The mean of three separately plated and treated plates is shown, with SD indicated by error bars. (C) Mitomycin C -induced radial chromosomes in the cells shown in A. The cells were exposed to 40 ng/mL mitomycin C for 48 hr. 100 metaphases per sample were analyzed. (D) Arrows indicate radial chromosomes in *POLN*-depleted 293FT cells exposed to mitomycin C. Scale bar: 10μm.

These results contrast with previous reports that siRNA directed against *POLN* causes an increased sensitivity to MMC and cisplatin [11, 12]. Therefore, we performed siRNA-mediated knockdown in human cells using the previously reported targeting sequence [11]. In that study, the efficiency of siRNA-mediated knockdown was assessed using an antibody raised against pol ν. We have developed ~80 Pol ν monoclonal antibodies and two polyclonal antibodies against pol ν -77 [37], and identified several of them that can immunoblot and immunoprecipitate overexpressed pol ν in human cells (e.g. Mab#40 in **S8 Fig**). However, we have not been able to detect endogenous pol ν with these or other antibodies, and so we turned to a different approach to monitor the efficiency of siRNA-mediated *POLN* knockdown. Pol ν was expressed in a doxycycline-inducible manner in 293T-Rex cells. We confirmed complete knockdown of tagged protein expression, showing that the *POLN*-specific siRNA construct very effectively inactivates *POLN* mRNA (**Fig 11A,** top panel). We also performed quantitative PCR assays for *POLN* and *HAUS3* as described [21] and detected significant reduction of doxycycline-induced *POLN* transcript (P = 0.01) as well as endogenous *POLN* transcript (P = 0.03) by the *POLN*-specific siRNA (**S9 Fig**). Note that only incomplete transcripts of POLN have been detected in 293T cells [21]. *HAUS3* expression was not affected by this siRNA (**S9 Fig**). Nevertheless, neither pol ν-depletion (siN) nor pol ν-overexpression (293T-REx(POLN) + Dox, siC) had any influence on mitomycin C sensitivity in human cells (**Fig 11B and S8 Fig**). It was also proposed that pol ν depletion caused an increased formation of radial chromosomes in human cells treated with MMC [11]. We therefore analyzed metaphase spreads of 293T-REx depleted of pol ν, and 293FT cells depleted of pol ν, *FANCA*, or *FANCD2* (**Fig 11A**). Suppression of *FANCA* and *FANCD2* resulted in an increased frequency of radial chromosomal formation after treatment with mitomycin C as expected for these Fanconi anemia gene products. However, pol ν depletion did not increase the frequency of radial chromosome formation (**Fig 11C and 11D**).

## Discussion

### Viability and lifespan of mice lacking pol ν

The investigations presented here answer many long-standing questions about pol ν function and expression. An analysis of mice with genetic disruptions of *Poln* has not been reported previously. Because *Poln* is expressed only in some metazoan lineages, many widely-used model organisms are not relevant for biological analysis of pol ν. We found that *Poln* is not essential for mouse embryonic development or for viability. Three independently established and targeted mouse models that disrupt *Poln* were fully viable, in C57BL/6J;129Sv and FVB/NCrl backgrounds. The *Poln* deficient mice have a normal lifespan and lack abnormalities for many investigated anatomic features. Consistent with this, the International Mouse Phenotyping Consortium (mousephenotype.org) has recently listed mice with a homozygous knockout of exon 6. No abnormalities in adults or embryos were detected in a battery of anatomical, physiological and behavioral tests. No expression of *Poln* was detected in embryos, also consistent with our reported observations [21].

### Evidence for a specialized function of pol ν in testis

Our observations point to a function of pol ν in the testis. We discovered that *Poln* is uniquely upregulated during mouse testicular development and that it is enriched in spermatocytes. This may be a conserved feature in organisms where POLN occurs. For example, we found that *POLN* expression was essentially undetectable in somatic adult cells or embryonic cells from the zebrafish, but was present in adult zebrafish testis [21]. Further, the phylogenetic analysis described here shows that *POLN* genes exist only in metazoan animals that use sexual reproduction and deploy sperm. This is consistent with a specialized function for pol ν in discrete spermatocyte-forming organs. The evolutionary distribution of *POLN* is a contrast to the wide distribution of *POLQ* genes in animals and plants, including many unicellular species and some that reproduce asexually. The DNA polymerase activity of pol ν appears to be critical for its function, as all organisms encoding the enzyme retain the six conserved DNA polymerase motifs with the residues necessary for catalytic activity. Expression of recombinant protein from the human, mouse and zebrafish *POLN* cDNAs all produce active DNA polymerase [21].

Importantly, it is uncertain whether the pol ν protein is expressed significantly in mammalian somatic cells or tissues. Most *POLN* transcripts in somatic human cells are inactive, alternatively spliced variants [3]. The protein is not detectable with the antibodies currently available, although mass spectrometry experiments detect a few peptides representing pol ν in various mouse, human, and rat tissues including brain, heart, kidney, liver, lung, pancreas, spleen, and testis [38]. The low level expression of mostly inactive transcripts is consistent with the lack of phenotypes in somatically derived cells, described here.

Pol ν may be important at specific recombination hot spots where DNA forms unique DNA secondary structures which provide challenges for other DNA polymerases. We detected a modest but significant reduction in meiotic recombination frequencies at the *A3* hot spot in *Poln* deficient mice. The frequency was lower in the crossover refractory zone of *A3* in *Poln* deficient mice. This zone harbors insertion/deletion polymorphisms and possibly forms a hairpin structure inhibiting replicative DNA polymerases [24]. The strand displacement activity of pol ν [4] may be effective in helping to synthesize the secondary structure-forming sequence. In fact, the crystal structure of pol ν reveals a cavity in the polymerase domain, which could accommodate a looped-out primer strand [6]. Such a looped-out primer could be formed during annealing of insertion/deletion polymorphisms during meiotic recombination.

Further, we previously reported proteins associated with pol ν when it was overexpressed in human cells. Mass spectrometry analysis showed associations of pol ν with homologous recombination factors including BRCA1, FANCJ, BRCA2, and PALB2 [21]. This is consistent with the participation of pol ν in a specialized homologous recombination reaction.

### Pol ν-defective mice and human cells have a normal response to DNA crosslinking agents

Our results show that mammalian cells have normal responses to DNA crosslinking agent exposure following pol ν elimination or depletion. This contrasts with the reported increased sensitivity to the DNA crosslinking agents MMC and cisplatin in human cells after an siRNA-mediated knockdown of *POLN* [11, 12],

Our conclusions are based on several independent knockouts of *Poln* function in the mouse, and on controlled siRNA and shRNA-depletion of *POLN* in human cells. These cells showed normal survival responses to crosslinks, and no evidence of increased frequency of MMC-induced radial chromosomes. We did not detect cellular crosslink-sensitivity after suppression of *POLN*. In studying pol ν, it is also crucially important to insure, as we have done here, that knockdown of *POLN* does not interfere with the expression of the essential *HAUS3* gene encoded in intron 1 of vertebrate *POLN*. Further, we emphasize that antibodies cannot be used currently to monitor endogenous knockdown of *POLN*. Finally, we have described that there is little evidence for expression of pol ν in somatic cells, which would explain the lack of phenotypes in non-germline cells when the gene is disrupted. Current evidence points towards a specific germ cell function.

Although pol ν shares homology with the DNA polymerase domain of *Drosophila* Mus308, which is involved in resistance to DNA crosslinking agents [9], it appears that a different Mus308 homolog is more involved in crosslink sensitivity in mammalian cells. HELQ, which shows similarity to the Mus308 helicase domain, is involved in DNA crosslink tolerance [34, 33, 35]. Our results are more in line with the lack of sensitivity to DNA crosslinking agents that was reported for *POLN*^−/−^ chicken DT40 cells [39, 40]. The ability of pol ν to perform limited bypass of a major groove DNA-peptide adduct (*N*^6^-A-peptide) and a DNA-DNA (*N*^6^-A-*N*^6^-A) crosslink residue [18] may be more broadly related to properties of pol ν that allow it to bypass blocks such as the secondary DNA structures that exist within meiotic recombination hotspots.

### Perspective

In many organisms, it is a common observation that knocking out a single DNA repair protein generates little or no DNA damage or growth-related phenotype. Double mutations, or disruption of backup pathways, are frequently necessary to reveal significant phenotypes. For example, no phenotype of pol ι disruption has been detected as a single mutant. In contrast, pol η /pol ι double-deficient mice show an altered spectrum of UV radiation-induced tumors [41, 42]. We constructed pol ν /pol θ double-defective mice, and as described here, this double mutation did not exacerbate cellular sensitivity to DNA damaging agents beyond what we observed with pol θ defective animals. Further screening of double mutants seems a possible approach to unveil the biological function of pol ν in vertebrates. The present work provides a major resource and foundation that paves the way for further exploration of the function of this unique human enzyme.

## Materials and methods

### Ethics statement

Research mice were handled according to the policies of the MD Anderson Cancer Center Institutional Animal Care and Use Committee, under approved protocol number 00001119-RN00.

### Targeting the *Poln*^ΔEx2^ allele disrupted from exon 2

The strategy for cre-loxP based knockout and targeting vector construction was designed and performed by genOway (Lyon, France). The genomic region of interest containing the murine *Poln* locus was isolated by PCR from genOway's 129Sv BAC library. PCR fragments were subcloned into the pCR4-TOPO vector (Invitrogen). The following primers were used. For the short homology arm: 5’-CATAACAGGTCAGAGTCACAAAACAGATATGC-3’and 5’- TATCTCACAGACATCAAAACCTACACATGCC-3’; for proximal long homology arm: 5’-CCAGGTAATTTAGATGTGTGAACCAGATGC-3’ and 5’- ACAAACTTTCCAGAACAAGGACAATGACC-3’; for distal long homology arm: 5’-GGGACAGAATAGAAACAAAATGACAAAATAGACC-3’ and 5’-GCTTATGCAAGTAGAGATTCAAAGTTGATGTAAGG-3’. The resulting sequenced clones (containing intron 1 to intron 2) were used to construct the targeting vector. A region including exon 2 was flanked in the adjacent introns by a Neo cassette (LoxP site—FRT site—PGK promoter—Neo cDNA—FRT site) and by a distal loxP site (**Fig 2A**). This allowed generation of constitutive and conditional knockout lines which had deleted the 145 bp *Poln* exon 2 containing the translational initiation codon, 760 bp of the upstream intron and 63 bp of the downstream intron. In the knockout, the deleted region is replaced by a 50 bp fragment containing the distal loxP site, simultaneously disrupting an endogenous AflII restriction enzyme site.

### Screening of *Poln* targeted ES cell clones

Linearized targeting vector was transfected into 129Sv ES cells (5 x 10^6^ ES cells in presence of 40 μg of linearized plasmid, 260 V, 500 μF). Positive selection was started 48 hr after electroporation, by addition of 200 μg/mL of G418. 2547 resistant clones were isolated and amplified in 96-well plates. Duplicates of 96-well plates were made. The set of plates containing ES cell clones propagated on gelatin were genotyped by both PCR and Southern blot analysis. For PCR analysis, one primer pair was designed to amplify sequences spanning the 5’ homology region. This primer pair designed to specifically amplify the targeted locus was: sense, 5’ GAAAAGCCTCGAAGATATGGGCACC-3', anti-sense (Neo cassette) 5'- GCCTCCCCTACCCGGTAGAATTAGATC-3'. Targeting was confirmed by Southern blot analysis using internal and external probes on both 3’ and 5’ ends. Two clones were identified as correctly targeted at the *Poln* locus.

### Generation of mosaic mice and breeding scheme

Clones were microinjected into C57BL/6J blastocysts, and gave rise to male mosaics with a significant ES cell contribution (as determined by an agouti / black coat color). Mice were bred to C57BL/6J mice expressing Flp recombinase to remove the Neo cassette (B6;129Sv-*Poln*^*tm1*.*1Rwd*^ designated as *Poln*^lox^ allele) and to female C57BL/6J mice expressing Cre recombinase to generate a germline deletion of Poln (B6;129Sv-*Poln*^*tm1*.*2Rwd*^ designated as *Poln*^ΔEx2^ allele). The following genotyping primers were used. *Poln*^lox^: forward, 5'- GAACCAGATGCTTGTTTGTTCTTTTCACC-3'; reverse, 5'- GTGTACTGAAATACTCCTCAGTTCTAAAAACGACC-3'; wild-type 152-bp product, floxed 266-bp product. *Poln*^ΔEx2^: forward, 5'- CAGGTAATTTAGATGTGTGAACCAGATGCTTG-3'; reverse, 5'- GGCAGTACAATAACAGAAACACTTCTCTTATGACC-3'; wild-type 1402-bp product, deleted 484-bp product (**S2 Fig**). Animals were validated by Southern blot analysis of AflII-digested DNA using a 5’ probe (**Fig 2**) of 364-bp probe for the Southern blot was generated by PCR on genomic DNA using the primer set: 5’-TGGGCAGTTAACTTAGTGGCAACTCTACT-3’ and 5’- GTGTACCTGATCTGTTCCATGTCTTCATATAATC-3’.

### Zinc-finger nuclease (ZFN) targeting

Sixteen ZFN pairs targeting *Poln* were designed and assembled by PCR subcloning into the pZFN plasmid by Sigma. All pairs were tested for efficiency of generating double strand breaks using the Surveyor Mutation assay in cultured mouse Neuro2A cells. The selected ZFN pair, targeting exon 21, was used for subsequent microinjections into FVB/NCrl embryos. The ZFN target site (cleavage site in lowercase) is: 5’-GGCCTCTCCCGAGGAtctgtGCACAGGACCAGCAG-3’. Validation used forward primer: 5’-CCCTGGGAATACTTGGGACT and reverse primer: 5’-ACTACCAGGCAGGACAGGTG.

Embryos were obtained from superovulated Charles River FVB/NCrl female mice. ZFN pairs were microinjected and transferred into pseudopregnant foster females. Thirty-six pups were born. The pups were sampled for genotyping at approximately 3 weeks of age. Four founders (11% efficiency) have been identified. Three of the four founders resulted in early stop codons. Founder #22 has a 4 base pair deletion (FVB/NCrl.Cg-*Poln*^*em1Rwd*^ designated as *Poln*^*del4*^ allele). Founder #26 has a 13 base pair deletion (FVB/NCrl.Cg-*Poln*^*em2Rwd*^ designated as *Poln*^*del13*^ allele) and #27 is chimeric with a 4 and 13 base pair deletion. An ApaL1 restriction site is present in the ZFN targeted site and the sequence is lost in the 4-bp and 13-bp-deleted allele. This is used to validate the genotyping result (**S3 Fig**).

### Mouse aging study

Thirty mice (15 males and 15 females) of each genotype (*Poln*^ΔEx2/ΔEx2^, *Poln*^+/ΔEx2^, and *Poln*^+/+^) were monitored over time for tumor incidence and survival. Mice were housed in an SPF modified barrier facility under standard light cycle and temperature conditions. Sentinal animals were free of multiple murine infectious and parasitic agents including *Helicobacter* and mouse parvoviruses. Mice were fed irradiated standard rodent diet (Harlan Laboratories Irradiated Teklad 22/5 Rodent Diet #8940) ad libitum and provided with reverse osmosis purified water via an automated system. Mice were monitored from approximately 3 weeks of age until they reached a moribund state. Mice were monitored daily for overall health status. Additionally, body condition score [43] and body weight were assessed weekly and recorded. Mice were removed from the study if any of the following criteria were met: Loss of greater than 20% body weight in one week, body condition score of 1 or 2 [43], respiratory or ambulatory difficulties, or non-healing dermatitis.

Mice removed from study were killed by carbon dioxide inhalation followed by cervical dislocation before complete necropsies were performed. Body weight and weights of the following organs were obtained: spleen, kidneys, liver, adrenals, testes, heart, thymus, and brain. All tissues were examined grossly, fixed for 24–48 hr in neutral buffered formalin, and stored in 70% ethanol. For all mice, H&E-stained slides of the following tissues were prepared by standard techniques and examined by a board-certified veterinary pathologist: kidneys, liver, heart, lungs, thymus, spleen, testes or ovaries, brain, all gross lesions. For all suspected lesions of histiocytic sarcoma or lymphoma, immunohistochemistry was performed to identify T cells (CD3), B-cell (CD45R), and macrophages (F4/80 or MAC). Additional immunohistochemistry was performed as required for specific diagnosis.

### Serum chemistry

Physiological serum parameters were measured using the VetScan VS2 Analyzer with the Comprehensive Diagnostic Profile reagent rotor (Abaxis).

### Cell culture

The primary mouse embryonic fibroblasts (MEFs) were derived from e13.5 embryos with genotypes *Poln*^+/+^, *Poln*^+/ΔEx2^ and *Poln*^ΔEx2/ΔEx2^ (C57BL/6J mouse, in which exon 2 encoding the first methionine was deleted) and *Poln*^+/+^, *Poln*^+/del4^ and *Poln*^del4/del4^ (FVB/NCrl mouse, in which a Zinc Finger Nuclease-mediated 4 bp frameshift was introduced in the DNA polymerase domain of *Poln*). Primary MEFs were cultured in medium containing high glucose, glutamax-DMEM (Invitrogen), 15% Hyclone FBS (Thermo Scientific), non-essential amino acids, sodium pyruvate, MEM vitamin solution, penicillin/streptomycin (Invitrogen) and maintained in air-tight containers filled with a gas mixture containing 93% N_2_, 5% CO_2_ and 2% O_2_ (Praxair) at 37°C. MEFs were immortalized with SV40 Tag as reported [36]. Immortalized MEFs were cultured in medium containing high glucose, glutamax-DMEM (Invitrogen), 10% FBS (Atlanta Biologics) and penicillin/streptomycin and maintained in a humidified 5% CO_2_ incubator at 37°C.

### Isolation of the mouse *Poln* cDNA

We prepared a cDNA library was prepared from 129Sv mouse testis. The full-length open reading frame (ORF) was amplified with primers (5’-caccaaaatggaaaattatgaggcatgtg and 5'-caggagatcctgggctcagccgactacaaagacgatgacgacaagtaggaattcatatat), and cloned into pENTR/D-TOPO vectors and then recombined into pDEST17 vectors (Invitrogen).

### Quantitative PCR (qPCR) assay

Total RNA was extracted using TRIzol (ThermoFisher Scientific), and RNA integrity assessed using the Agilent 2100 bioanalyzer (Agilent Technologies, Inc.). Total RNA (1 μg) was used as template to synthesize cDNA with the High-Capacity cDNA Reverse Transcription Kit (Applied Biosystems). qPCR was then performed on the ABI 7900HT Fast Real-Time PCR System (Applied Biosystems). Custom assays for mouse *Poln*, *Polq*, and *Pold1* were designed using FileBuilder 3.1 software (Applied Biosystems) and ordered from Applied Biosystems. TaqMan primer and probe sets for each gene are shown in **Table 6**. The absolute quantity (AQ) of transcripts for *Poln* and *Polq* was determined using the generated standard curves and Applied Biosystems’ Sequence Detection Software version 2.2.2 (ABI). Standard curves for each gene were determined using the plasmids pDEST17 carrying cDNA coding full length of *Poln*, and *Polq* clone (MGC:189905, IMAGE:9088092). For relative quantification, TaqMan primers and the probe set for *Gapdh* were purchased from Applied Biosystems. Triplicate qPCR reactions each containing cDNA representing 40 ng of reverse-transcribed total RNA were then assayed for transcript quantity with *Gapdh* serving as endogenous controls to normalize input RNA levels.

**Table 6:** TaqMan primer and probe sets for mouse.

	TaqMan primer	TaqMan probe
*Poln*	5’- GATGTACAAGGATGGTTCCACACA 5’- GCCTACATGGCTTTTTAGTAACACTACAAT	5’- AAAGCCTCCTTGGCGCTCA
*Polq*	5’- GGCTCTGAAGAACTCTTTGCCTTT 5’- GCTGCTTCCTCTTCTTCATCCA	5’- TCCGGGCACTTTTG
*Pold1*	5’- TTTGACCTCCCATACCTCATCTCT 5’- ACGCGGCCCAGGAAAG	5’- AAGCGGTCCACCTTTAG
*Haus3*	5’- CTCCCATCGACACCAAAGATCA 5’- CAGGTGTTCATGGGTTATAAACAATTCTT	5’- CATAGGCTTTATGAACTTCT

### Preparation of spermatogenic cells

Cell suspensions were prepared from 25 adult mice (~100 days old) by previously published methods [44, 45]. Seminiferous tubules were isolated by incubating the decapsulated testes with collagenase (0.5 mg/mL) and DNase I (200 μg/mL) in enriched DMEM/F12 (Invitrogen), to which 0.1 mM non-essential amino acids (Invitrogen), 1 mM sodium pyruvate (Invitrogen), and 5 mM sodium lactate (Sigma) were added. This decapsulated testis tissue was shaken for 15 min at 35°C in a water bath, until it was mostly dispersed into tubules. The dispersed tubules were allowed to settle and, after removal of the supernatant, were resuspended in 32 mL of DMEM/F12 solution. In each of four 50 mL tubes, 8 mL of this suspension was layered onto 40 mL of 5% Percoll solution (Sigma), and the tubules were allowed to settle until most of the larger tubules and clumps were at the bottom. The supernatants were removed and the settled tubules were washed with DMEM/F12 solution and then further digested with trypsin (1 mg/mL) and DNase I (200 μg/mL) in enriched DMEM/F12 for 20 min at 35°C with shaking. Fetal bovine serum was added to 10%, and the cells were dispersed by pipetting. Total cell suspensions were separated by centrifugal elutriation (JE-6B rotor, Beckman) to obtain fractions enriched in spermatids (flow rate: 15.6 mL/min, rotor speed: 2250 rpm) and pachytene primary spermatocytes (37 mL/min, 2250 rpm). The purified pachytene fraction was obtained by plating the Percoll-enriched fraction on DSA (Sigma) coated dishes, to which Sertoli cells bind strongly, and the pachytene spermatocytes were recovered in the unbound cell fraction. The Sertoli cells recovered from the elutriator were purified by plating them on the DSA-coated dishes, removing the unbound and loosely bound cells, and directly extracting the bound Sertoli cells with RLT lysis buffer (QIAGEN). The purity of each fraction was initially determined by cell smears stained with periodic acid Schiff-hematoxylin.

### Testis morphology

Testes were removed from killed mice and fixed in neutral-buffered formalin for 24 hr before being processed for histologic sectioning (4 μm) and stained with hematoxylin and eosin (H&E). The samples were analyzed using a BX41 Olympus microscope with 10X objective.

### Generation of F1 hybrid mice

In order to detect recombinant molecules at the *A3* locus in sperm, F1 hybrid animals carrying heterozygous *A3* alleles were generated. The *Poln*^ΔEx2^ allele in the C57BL/6J background and the *Poln*^*del4*^ allele in the FVB/NCrl background were backcrossed into DBA2/J strain. The C57BL/6J or FVB/NCrl strains share an ~1.8% polymorphism density at *A3* with the DBA2/J strain allowing detection of recombinant molecules from sperm DNA. F1 hybrid males were generated from matings of parents carrying each of the *Poln* mutant alleles in one of the background strains.

### Spermatocyte spreads and immunofluorescence

Spermatocyte spreads were prepared according to previously published protocol [46]. Briefly, slides rinsed with PBS were blocked for 30 min at room temperature in Gelatin Block Solution (GBS: 0.2% v/v fish gelatin (Sigma G-7765), 0.2% v/v IgG-free BSA (Jackson ImmunoResearch 001-000-162), 0.05% w/v Tween-20). Next, covered slides were incubated overnight at 4^°^C with 100 μl of primary antibody diluted in GBS. Slides were then washed with GBS on a rotating platform shaker (one quick rinse followed by 5, 10 and 15 min rinses). Next, covered slides were incubated for 45 min at 37°C with 100 μl of secondary antibody diluted (1:200) in GBS. Slides were washed with GBS as above, followed by two 5 min washes with 0.4% PhotoFlo 200 (Kodak 1464510). Slides were then dried in the dark and mounted with Prolong^®^ Gold antifade with DAPI. Antibodies were used in two combinations in this study (dilution): Rabbit anti-SCP3 (1:500; Santa Cruz Biotechnology sc-33195) and Mouse anti-MLH1 (1:20; BD Pharmingen 551092) or Mouse anti-SCP3 (1:200; Santa Cruz Biotechnology sc-74569); and Rabbit anti-SCP1, (1:200; Abcam ab15090); secondary antibodies (all 1:200) were: Goat anti-Rabbit 594 (Life Technologies A11037) and Goat anti- Mouse 488 (Life Technologies A11029) or Goat anti-Mouse 594 (Life Technologies A11029) and Goat anti-Rabbit 488 (Life Technologies A11034). Images were acquired on a Zeiss Axio Imager M2 with a Plan-Apochromat 100x/1.4 oil immersion objective.

### DNA isolation and determination of amplifiable DNA concentration

DNA samples were isolated from sperm from epididymides of adult (2–5 m.o.) F1 hybrid animals, as previously described [24, 25]. Two animals were analyzed for each allele, per genotype. Briefly, a standard phenol/ chloroform/ isoamyl alcohol DNA isolation protocol was followed by ethanol precipitation. An aliquot of each DNA sample was used to quantify DNA concentration by UV absorbance and by comparison to a dilution series by agarose gel electrophoresis, using high quality sperm DNA of defined concentration. The number of amplifiable DNA molecules/pg was determined by performing 12–24 PCR reactions per sample seeded with 12 pg per reaction (equivalent to 2 amplifiable molecules/well).

### Crossover assay

The crossover assay for *A3* was as described previously [24, 25]. Briefly, recombinant molecules were identified after two rounds of nested PCR by 0.8% agarose gel electrophoresis and positive reactions (putative crossovers) were then PCR-amplified, together with positive and negative controls. Products in a 96-well format were dot-blotted onto nylon hybridization membrane (Roche, 11417240001) and genotyped by Southern blotting with allele-specific oligonucleotide (ASO) probes. Somatic DNA from spleen or liver for each assayed animal was used as a negative control at total DNA inputs equivalent to or higher than sperm DNA. No crossovers were detected in somatic controls (frequency < 1.05 x 10^−5^).

### Genotyping with ASO probes

A detailed version of the Southern blotting protocol for the *A3* hotspot has been described [24, 25]. Briefly, ASO probes were radiolabeled using T4 polynucleotide kinase and hybridized with nylon membranes containing PCR products from the crossover assay. After hybridization, blots were exposed for 4–5 hr on phosphorimager screens and scanned using the Typhoon FLA 9500 phosphorimager (GE Healthcare). Scans were then scored and positive signals used to generate crossover breakpoint maps. Each ASO is an 18-bp oligonucleotide designed to specifically hybridize with one of the two parental genotypes. At the *A3* locus, most of the FVB/NCrl sequence is identical to the C57BL/6J strain.

### Induction and repair of radiation-induced γ-H2AX foci in round spermatids

Wild-type and *Poln*^ΔEx2/ΔEx2^ mice were generated from *Poln* heterozygous knockout crosses, and *Polq*^-/-^ mice were generated from *Polq* heterozygous knockout crosses. 8–12 week-old mice received whole-body irradiation with 1 Gy or 2 Gy at 2 Gy/min, 160 kV peak energy (Rad Source 2000 irradiator, Suwanee, GA). For DSB induction, three wild-type mice per dose were analyzed at 30 min post-irradiation. For the DSB repair kinetics three mice per time-point were analyzed at 0.5, 5, 24, and 48 hr after irradiation with 2 Gy. In each experiment three mock-irradiated mice served as controls. After animals were sacrificed, testes were immediately removed and placed in fixative. Formalin-fixed tissues were embedded in paraffin and sectioned at an average thickness of 4 μm. Tissue sections were incubated with primary antibody against γ-H2AX (Bethyl) followed by Alexa Fluor-488-conjugated secondary antibody (Invitrogen). Finally, sections were mounted in VECTASHIELD mounting medium with 4′,6-diamidino-2-phenylindole (DAPI) (Vector Laboratories). For quantitative analysis, radiation-induced foci were counted by eye using a Leica DMI 6000 microscope equipped with a 63X oil objective. Tissue sections were incubated with primary antibody against γ-H2AX. To evaluate potential differences in DSB repair kinetics, the two-way ANOVA test was performed for each dose and repair-time. The criterion for statistical significance was p ≤ 0.05.

### RNA-seq analysis

For RNA-seq analysis, four biological replicates were prepared for *Poln*^+/+^ and *Poln*^ΔEx2/ΔEx2^ mice. RNA was purified from testis of 68-day old male mice with RNeasy kit (QIAGEN) with on-column DNase treatment. The libraries were prepared using the Illumina TruSeq stranded total RNA kit according to the manufacturer’s protocol, except that the PCR amplification cycle was reduced to 10. The libraries were sequenced on HiSeq 2000 (Illumina), generating 53–73 million pairs of 75 bp reads per sample. Each pair of reads represents a cDNA fragment from the library. The reads were mapped to mouse genome (mm10) by TopHat (Version 2.0.7) [47]. By reads, the overall mapping rate is 90–96%. 83–93% fragments have both ends mapped to mouse genome. The number of fragments in each known gene from RefSeq database 48 (downloaded from UCSC Genome Browser on March 6, 2013) was enumerated using htseq-count from HTSeq package (version 0.5.3p9) [48]. Genes with less than 10 fragments in all the samples were removed before differential expression analysis. The differential expression between conditions was statistically accessed by R/Bioconductor package edgeR (version 3.0.8) [49]. Genes with false discovery rate ≤0.05 and fold change ≥2 were called significant. Gene clustering and heatmap were done by Cluster 3.0 and TreeView. GO analysis was performed using Ingenuity Pathway Analysis (IPA) software. The transcriptional profiling plot (**Fig 7A**) was made using R software.

### Reticulocyte micronuclei

Approximately 100 μL of blood obtained from individual mice by cardiac puncture were collected into tubes containing 350 μL of heparin solution and fixed in ultra-cold methanol according to the protocol in the Mouse MicroFlowBasic Kit (Litron Laboratories). The fixed samples were stored at −80°C until the flow cytometry analysis was performed. Methanol-fixed blood samples were washed and labeled with anti-CD71-FITC, anti-CD61-PE and PI for high speed flow cytometry using CellQuest software, v5.2 (Becton Dickinson, San Jose, CA). For each sample, 2 × 10^4^ CD71-positive reticulocytes were analyzed for the presence of micronucleated reticulocytes. Flow cytometers were calibrated by staining Plasmodium berghei-infected rodent blood (malaria biostandards) in parallel with test samples on each day of analysis. Statistical analysis was performed using the Student's t-test or one-way ANOVA followed by Tukey's test.

### B cell culture and CSR analysis

B cells were isolated from mouse spleens, purified by negative selection with anti-CD43 depletion (Miltenyi) and stimulated with IL-4 and Lipopolysaccharide and IL-4 (Sigma) for 72 hr. Where indicated, cultures were incubated with DNA-PKcs inhibitor 20 μM NU7026 (Tocris) dissolved in DMSO, or mock-treated. Three mice were analyzed in triplicate and cell count numbers and viability were similar for all groups. The culture, flow-cytometric analysis for CSR analysis and junction analysis has been described [32, 50]. Sμ-Sγ1 CSR junctions were amplified by PCR using the following conditions for 25 cycles at 95°C (30 s), 55°C (30 s), 68°C (180 s) using the primers (FWD 5′-AATGGATACCTCAGTGGTTTTTAATGGTGGGTTTA-3′; REV 5′ CAATTAGCTCCTGCTCTTCTGTGG-3′) and Pfu Turbo (Stratagene). To the PCR reaction, 5 U of Taq polymerase (Promega) was added and incubated at 72°C for 10 min. The resulting product was TOPO TA cloned and transformed into Top10 E. coli cells (Life Technologies) and plasmids were purified and sent for sequencing using M13 FWD and REV primers in addition to the amplification primers for sequencing. 100 clones for each group were analyzed for mutations, deletions, insertions, and sequence overlaps at the junction and both 30 nt upstream and downstream of the junction.

### Generation of stable cell lines

T-REx 293 cells (Invitrogen) were transfected with the pcDNA5/FRT/TO TOPO TA construct. Full-length pol ν with six His residues at the N-terminus and a FLAG tag at the C-terminus [4, 21] was inserted into the plasmid vector. Stable clones were selected with hygromycin and blasticidin. Expression of the Flag-tagged pol ν was induced with 0.1 μg/mL doxycycline (SIGMA) for 24 hr. Basal repression and doxycycline-induced expression of pol ν were confirmed by immunoblotting.

### siRNA transfection

The *POLN*-specific RNAs (designated ‘siN’, 5’- AAGCACCCAAUUCAGAUUACU) (Dharmacon), the FANCA-specific RNAs (designated ‘siA’, 5’-AAGGGUCAAGAGGGAAAAAUA-3’) (Invitrogen), the FANCD2-specific Stealth RNAs (designated ‘siD2’, 5’-CCAUGUCUGCUAAAGAGCGUUCAUU-3’) (Invitrogen) and ON-TARGETplus Non-Targeting siRNAs as a negative control designated ‘siC’ (Thermo Scientific) were used. The siRNAs were introduced into 293FT or 293T-REx *POLN* cells. 24 hr prior to transfection, cells were plated in a 6-well plate at 2.0 x 10^5^ cells/well. For each well, 5 pmol of siRNAs was diluted into 250 μl of Opti-MEM (Invitrogen). In a separate tube, 5 μl of Lipofectamine RNAiMAX reagent (Invitrogen) was diluted into 250 μl of Opti-MEM and incubated at room temperature for 10 min. The Lipofectamine RNAiMAX dilution was added into the diluted siRNA duplex and incubated at room temperature for 20 min. Before the transfection, medium was replaced with fresh 2.5 mL of DMEM supplemented with 10% fetal bovine serum for each well. The Lipofectamine RNAiMAX-siRNA complex was added dropwise to the cells and incubated at 37°C. After 24 hr the cells were washed, trypsinized, and plated with fresh DMEM medium supplemented with 10% fetal bovine serum and 1% penicillin-streptomycin (Invitrogen). To measure the levels of proteins, whole cell crude extracts were prepared 48 hr after the RNA transfection and analyzed by immunoblotting with anti-pol ν (PA434)[3], anti-FANCA (Bethyl, A301-980A, 1:10,000 dilution), anti-FANCD2 (GeneTex Inc., EPR2302, 1:2000 dilution), and anti-α-tubulin (Sigma, T5168, 1:8000 dilution) antibodies.

### shRNA transfection

The following sequence was cloned into pSIF-H1-copGFP vector to target *POLN*: 5’-GATCCTCTTTGGCGAGTTAGAGCTGTACTTCCTGTCAGATGCAGCTTTAACTTGCCAAAGAGGATTTTTT-3’, underlined sequence indicates the target sequence (System Biosciences). Lentiviral particles were generated by transfection of three plasmids (the expression plasmid, e.g., pSIF-H1-copGFP-hPOLN.1; plus pFIV-34N and pVSV-G) into 293FT cells using FuGene 6. Culture media from transfected cells was collected 48 hr after transfection to isolate the viral particles, passed through 0.45 μm filters, used immediately or stored at −80°C in single-use aliquots. Lentiviral transduction was completed as follows: Briefly, 6.0 × 10^4^ cells were seeded into 6-well plate and incubated for 24–30 hr at 5% CO_2_ at 37°C. Cells were transduced for 18 hr with shRNA-expression lentiviral stocks at 32°C and cultured for 72 hr at 37°C as described [51]. Stable cell lines were selected by detecting GFP expression (a co-expressed marker gene). To measure the levels of proteins, immunoblotting was performed with anti-pol ν (Mab#40) and anti-PCNA (Santa Cruz, sc-56, 1:1,000 dilution) antibodies.

### Survival experiments

For the ATPlite assay (PerkinElmer), 1,250 cells were plated per well into white 96 well plates and incubated twenty-four hr prior to inducing DNA damage. The cells were incubated with DNA damage inducing agents for the indicated time. After incubation, the cells were immediately lysed and assayed for ATPlite luminescence as described in the manufacturer's instructions. For the clonogenic assay, 1.0 x 10^5^ cells were plated in 60 mm culture plates and incubated for 24 hr prior to DNA damage induction. Groups of plates were exposed to indicated doses of mitomycin C for 1 hr. After making a dilution series for each group, cells were returned to the incubator until colonies could be detected in the samples (7 to 14 days), and then were fixed, stained, and scored for survival.

### Chromosome analysis

Cells were treated with 40 ng/mL of mitomycin C for 48 hr. At forty-four hr after mitomycin C treatment, cells were treated with 0.03μg/mL colcemid solution (Sigma) for 4 hr. The cells were then trypsinized and exposed to 0.075M KCl for 15 min at 37°C, and were fixed in 3:1 methanol:glacial acetic acid. The cells were spread on glass slides, Giemsa stained and metaphases were analyzed using a BX41 Olympus microscope, with 60X or 100X oil objectives. Photographs were taken with the 60X oil objective on a Spot Idea 5 color digital camera. 100 metaphases per sample were analyzed to identify cell population with radial chromosome.

## Supporting information

S1 Fig Sequence alignment of the DNA polymerase domain region of *POLN* homologs and prokaryotic A-family DNA polymerases. The six motifs conserved in family A DNA polymerases are indicated, as well as three insertions conserved in *POLN*, defined previously [5]. The alignment was generated using ClustalW in MacVector version 15.1.4. Similarity groups for shaded residues are: (K, R, H), (D, E), (A, G, I, L, V), (F, Y, W), (Q, N), (S, T), (C, M), (P). Representative sequences were used from the mammals *Homo sapiens* (human) and *Mus musculus* (mouse); the fish *Danio rerio* (zebrafish) and *Oreochromis niloticus* (tilapia); a bird *Zonotrichia albicollis* (white-throated sparrow); the echinoderm *Strongylocentrotus purpuratus* (sea urchin); two gastropods *Lottia gigantia* (owl limpet) and *Aplysia californica* (sea hare); the brachiopod *Lingula anatina*; the ctenophore *Mnemiopsis leidyi* (comb jelly); and the sponge *Amphimedon queenslandica*. The lower sequences are Pol I genes from bacteria: Ec, *Escherichia coli*; Pa, *Pseudomonas aeruginosa*; Re, *Rhodococcus erythropolis*; Taqpol, *Thermus aquaticus*; BspolI, *Bacillus subtilis*, RhpolI, *Rickettsia helvetica*.
(TIF)

S2 Fig Genotyping of the *Poln*^lox^ and *Poln*^ΔEx2^ mouse lines. (A) Agarose gel showing typical genotype results with *Poln*^ΔEx2^ primers. PCR of the wild-type allele yields a 1402-bp product, and the Cre-deleted allele yields a 484-bp product. (B) Agarose gel showing typical genotype results with *Poln*^lox^ primers. PCR of the wild-type allele produces a 152-bp product and the floxed allele a 266-bp product. The Cre-deleted allele does not amplify with the *Poln*^lox^ primers. (C) Diagram of the targeted mouse *Poln* allele, with the wild-type (*Poln*^+^) locus shown at the top. The second exon encoding the first methionine is indicated as a black box. The middle diagram represents the targeted allele (*Poln*^lox^) after Flp-mediated excision of the neomycin positive selection cassette. FRT sites are represented by double red triangles and loxP sites by blue triangles. The bottom diagram represents the targeted allele (*Poln*^ΔEx2^) after Cre-mediated excision of the wild-type exon 2. Locations of *Poln*^ΔEx2^ primers (blue) and *Poln*^lox^ primers (red) and expected product sizes are shown for each allele.
(TIF)

S3 Fig PCR and restriction enzyme-based genotyping for zinc finger nuclease targeted mice. (A) Polyacrylamide gel showing typical genotype results for wild-type and *Poln*^del4^ alleles with *Poln* Cel1 primers. The wild-type allele is a 376-bp product and the *Poln*^del4^ allele product is indistinguishable in size from the wild-type allele. Digestion of the wild-type *Poln* allele with the restriction enzyme ApaL1 yields 205-bp and 171-bp PCR products. Due to ablation of the ApaL1 restriction site, the *Poln*^del4^ allele product is resistant to digestion. *Denotes heteroduplex product. (B) Polyacrylamide gel showing typical genotype results for wild-type and *Poln*^del13^ alleles with *Poln* Cel1 primers. The wild-type allele is a 376-bp product and the *Poln*^del13^ allele product is indistinguishable in size from the wild-type allele. The *Poln*^del13^ allele product is also resistant to ApaL1 digestion. *Denotes heteroduplex product. (C) Diagram of the ZFN targeted mouse *Poln* allele, with the wild-type (*Poln*^+^) allele shown at the top. The blue arrow indicates the ApaL1 restriction site. The middle diagram represents the *Poln*^del4^ targeted allele. The red bar denotes the location of the 4-bp deletion. The bottom diagram represents the *Poln*^del13^ targeted allele. The red bar denotes the location of the 13-bp deletion. Locations of *Poln* Cel1 primers and expected full-length product sizes are shown for each allele.
(TIF)

S4 Fig Reciprocal crossover asymmetry at the *A3* hotspot in the FVB/NCrl x DBA/2J background suggests reduced meiotic DSBs on the FVB/NCrl *A3* allele. (**A**) Top, crossover breakpoint maps from FVB/NCrl (F) x DBA/2J (D) in the F-to-D (black) and D-to-F (gray) orientations for *Poln*^+/del4^. Bottom, cumulative distribution fraction (CDF) of crossover breakpoints. The dashed vertical lines represent the average midpoint of crossover breakpoints in each orientation. Note that the midpoints in the F-to-D and D-to-F orientation are shifted relative to each other, indicating strong reciprocal crossover asymmetry. The asymmetric distribution in crossover breakpoints is interpreted to reflect biased DSB formation in favor of one of the parental chromosomes, in this case D. (**B**) Top, crossover breakpoint maps from C57BL6/J (B) x D in the B-to-D (black) and D-to-B (gray) orientations for *Poln*^+/ΔEx2^. Bottom, CDF of crossover breakpoints showing mild reciprocal crossover asymmetry. (**C**) Similar to (A): Top, crossover breakpoint maps in the F-to-D (red) and D-to-F (orange) orientations for *Poln*^del4/del4^. Bottom, strong reciprocal crossover asymmetry is observed in the F x D background. (**D**) Similar to (B): Top, crossover breakpoint maps in the B-to-D (red) and D-to-B (orange) for *Poln*^ΔEx2/ΔEx2^. Bottom, mild reciprocal crossover asymmetry is observed in the B x D background. (**E**) The binding of a meiosis-specific zinc finger protein, PRDM9, dictates the location of most meiotic DSBs in the mouse. There is a PRDM9 binding motif at the center of the *A3* locus [26]. The consensus PRDM9 binding motif derived by Brick et al. [52] is also indicated. The binding motif sequence differs between strain backgrounds, which affects the affinity of PRDM9 binding [26] and likely the frequency of meiotic DSBs. The yellow bar and yellow shading represent the predicted PRDM9 binding site. Red arrowheads indicate polymorphisms implicated in differential PRDM9 binding. The PRDM9 binding motif in FVB/NCrl is predicted to have lower affinity than the motif in the A/J background. It was previously shown that in A/J x DBA/2J F1 hybrids, the *A3* hotspot has a strong reciprocal crossover asymmetry [24], similar to that observed in F x D. Taken together, the reduced recombination frequency in F x D as compared to B x D mice can be explained by reduced DSBs on the FVB/NCrl *A3* allele.
(TIF)

S5 Fig Meiotic recombination in *Poln* deficient mice. Total crossover breakpoints found in 337 *Poln* heterozygote controls (157 *Poln*^+/del4^ and 180 *Poln*^+/ΔEx2^) and 340 *Poln* knockouts (155 *Poln*^del4/del4^ and 185 *Poln*^ΔEx2/ΔEx2^) are shown. Numbers of crossovers examined, Poisson-adjusted frequencies (± SD), and P-values (Fisher’s exact test) are indicated. Ticks represent positions of the tested polymorphisms. Arrows, insertion/deletion polymorphisms.
(TIF)

S6 Fig The inactivation of pol ν does not sensitize primary mouse embryonic fibroblasts to DNA damaging agents. Cells were exposed to indicated doses of mitomycin C for 72 hr (A), olaparib for 72 hr (B), etoposide for 72 hr (C) and 5-FU for 72 hr (D). *Poln*^+/+^ primary MEFs (circles), *Poln*^+/del4^ primary MEFs (triangles), *Poln*^del4/del4^ primary MEFs (cross), *Rev3L*^+/+^*;p53*^*−/−*^ SV40 Tag-immortalized MEFs (lozenge) and *Rev3L*^-/-^*;p53*^*−/−*^ Tag-immortalized MEFs (square). Viability was determined by measuring ATP content as described in Materials and Methods. The mean of three separately plated and treated experiments is shown, with SD indicated by error bars.
(TIF)

S7 Fig *Polq* deletion does not influence the sensitivity of *Poln* deficient MEFs to bleomycin and mitomycin C. MEFs were exposed to indicated doses of bleomycin for 24 hr and incubated for 72 hr (A) and mitomycin C for 48 hr (B). *Poln*^+/+^ *Polq*^+/+^: wild-type, *Poln*^ΔEx2/ΔEx2^2 and *Poln*^ΔEx2/ΔEx2^5: individual *Poln* knockout, *Polq*^-/-^: *Polq* knockout, *Poln*^ΔEx2/ΔEx2^ *Polq*^-/-^ 2,3, and 5: individual *Poln Polq* double knockout. All MEFs were SV40 Tag-immortalized. Viability was determined by measuring ATP content as described in Materials and Methods. The mean of three separately plated and treated experiments is shown, with SD indicated by error bars.
(TIF)

S8 Fig shRNA mediated pol ν knockdown does not sensitize human cells to mitomycin C. (A) Upper panel: immunoblot showing efficacy of shRNA-mediated knockdown of POLN (shN) in 293T-REx doxycycline inducible POLN cells. shC served as a negative control and PCNA as loading control. Monoclonal anti-pol ν antibody (Mab#40) recognized overexpressed pol ν but not endogenous pol ν. (B) Cell survival determined by using clonogenic survival assays. The mean of two independent experiments is shown, with SE indicated by error bars.
(TIF)

S9 Fig Relative amount of *POLN and HAUS3* mRNA after doxycycline induction and RNAi transfection. The efficacy of siRNA-mediated knockdown of *POLN* (siN) in 293T-REx doxycycline inducible *POLN* cells. The TaqMan primers spanned across adjacent exons of the human gene as described [21]. siC served as a negative control. *HAUS3* was analyzed simultaneously. Note that full-length POLN is not appreciably expressed in 293T cells, although partial transcripts representing portions of the mRNA can be detected [21]. To evaluate the extent of the reduction, a paired t-test was performed.
(TIF)

S1 Table List of differentially expressed genes in *Poln*^ΔEx2/ΔEx2^ mouse testes compared to *Poln*^+/+^ mouse testes. (XLSX)

S2 Table Assay for CSR from IgM to IgG1. (DOCX)
