# Supplementary figures and images for: Analysis of DNA polymerase ν function in meiotic recombination, immunoglobulin class-switching, and DNA damage tolerance
Source: PLoS Genet. 2017 Jun 1;13(6):e1006818. doi: 10.1371/journal.pgen.1006818 (PMC5472330; doi:10.1371/journal.pgen.1006818)

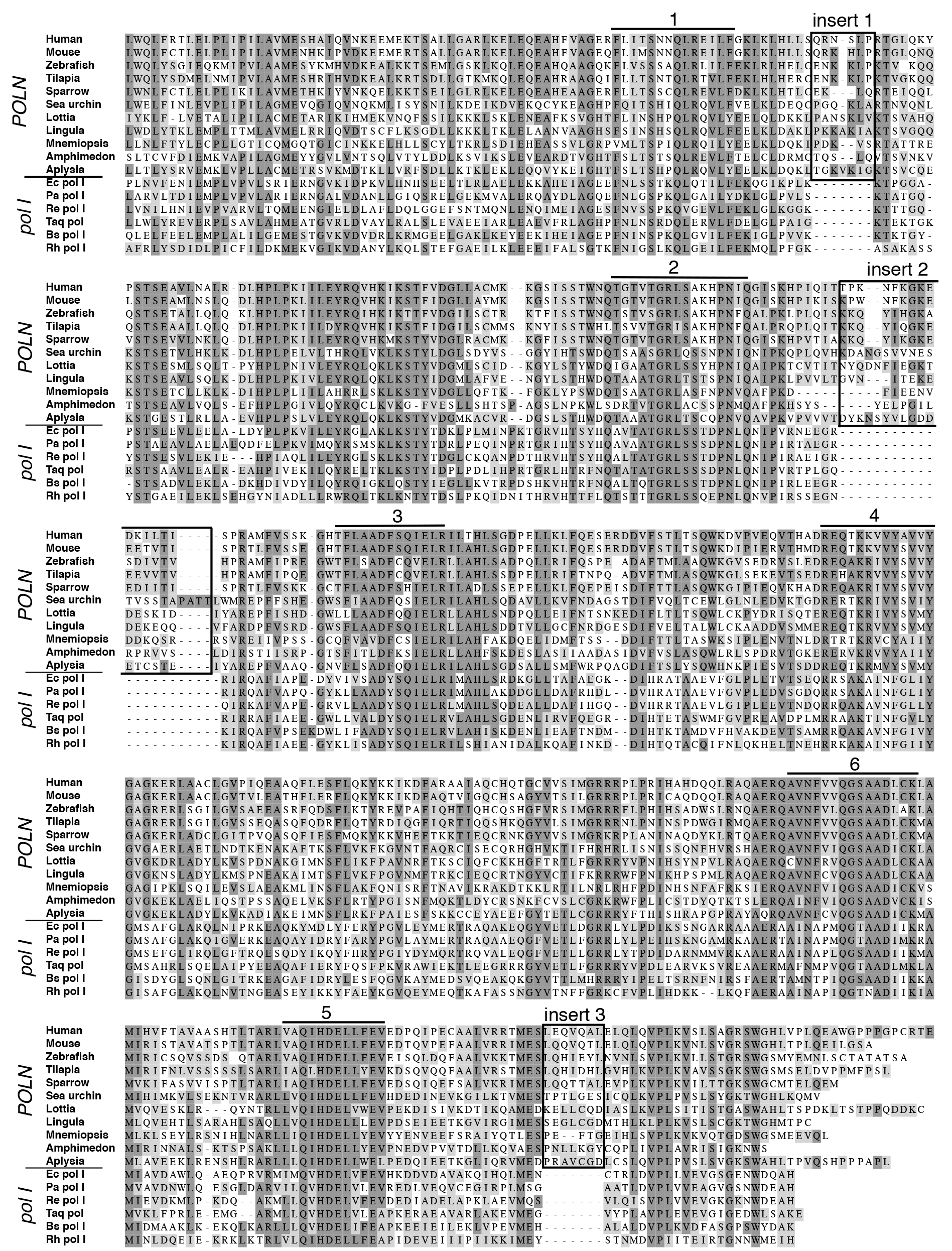

Supplement: S1 Fig — The six motifs conserved in family A DNA polymerases are indicated, as well as three insertions conserved in POLN, defined previously [5]. The alignment was generated using ClustalW in MacVector version 15.1.4. Similarity groups for shaded residues are: (K, R, H), (D, E), (A, G, I, L, V), (F, Y, W), (Q, N), (S, T), (C, M), (P). Representative sequences were used from the mammals Homo sapiens (human) and Mus musculus (mouse); the fish Danio rerio (zebrafish) and Oreochromis niloticus (tilapia); a bird Zonotrichia albicollis (white-throated sparrow); the echinoderm Strongylocentrotus purpuratus (sea urchin); two gastropods Lottia gigantia (owl limpet) and Aplysia californica (sea hare); the brachiopod Lingula anatina; the ctenophore Mnemiopsis leidyi (comb jelly); and the sponge Amphimedon queenslandica. The lower sequences are Pol I genes from bacteria: Ec, Escherichia coli; Pa, Pseudomonas aeruginosa; Re, Rhodococcus erythropolis; Taqpol, Thermus aquaticus; BspolI, Bacillus subtilis, RhpolI, Rickettsia helvetica. (TIF) [file pgen.1006818.s001.tif]

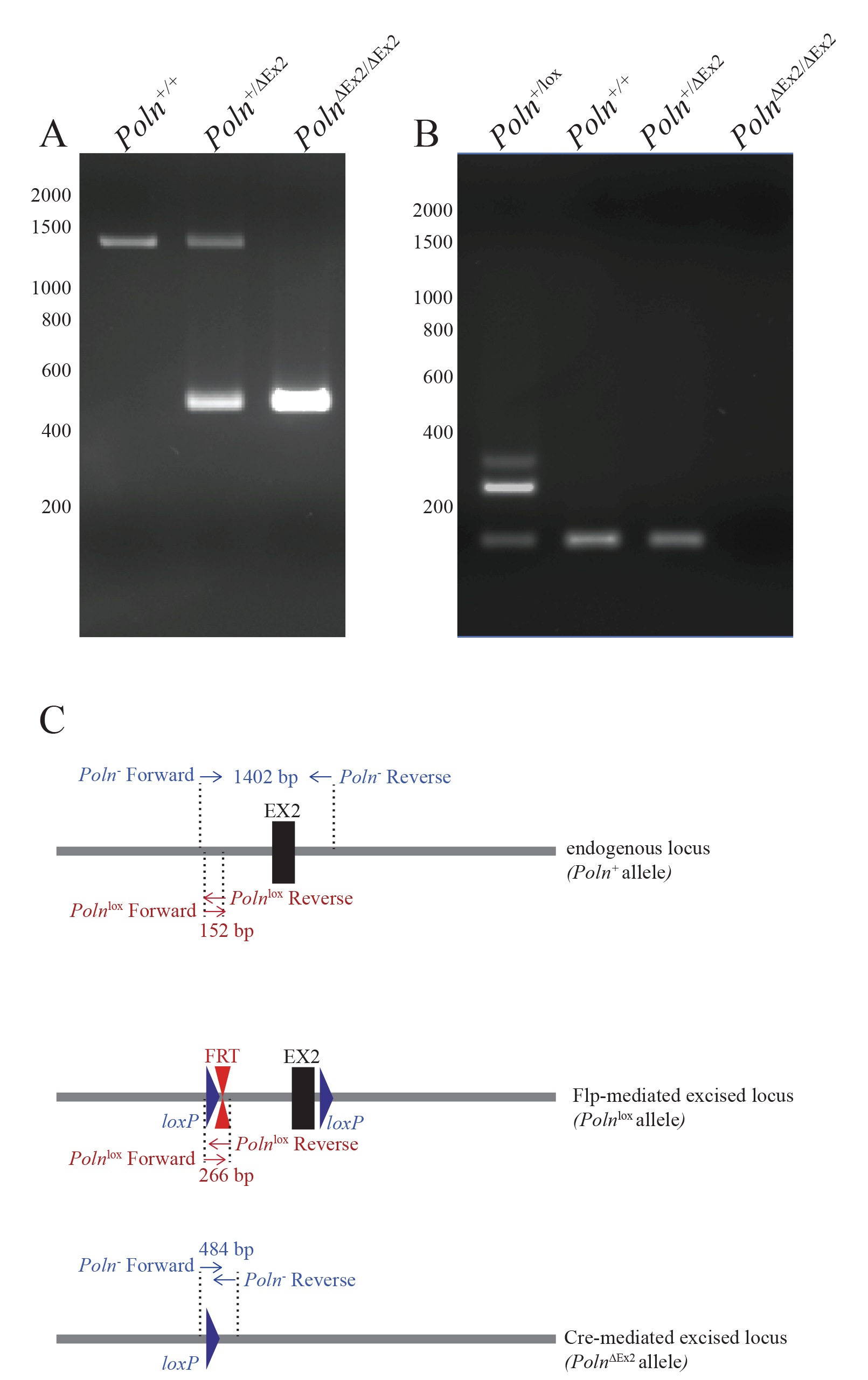

Supplement: S2 Fig — (A) Agarose gel showing typical genotype results with PolnΔEx2 primers. PCR of the wild-type allele yields a 1402-bp product, and the Cre-deleted allele yields a 484-bp product. (B) Agarose gel showing typical genotype results with Polnlox primers. PCR of the wild-type allele produces a 152-bp product and the floxed allele a 266-bp product. The Cre-deleted allele does not amplify with the Polnlox primers. (C) Diagram of the targeted mouse Poln allele, with the wild-type (Poln+) locus shown at the top. The second exon encoding the first methionine is indicated as a black box. The middle diagram represents the targeted allele (Polnlox) after Flp-mediated excision of the neomycin positive selection cassette. FRT sites are represented by double red triangles and loxP sites by blue triangles. The bottom diagram represents the targeted allele (PolnΔEx2) after Cre-mediated excision of the wild-type exon 2. Locations of PolnΔEx2 primers (blue) and Polnlox primers (red) and expected product sizes are shown for each allele. (TIF) [file pgen.1006818.s002.tif]

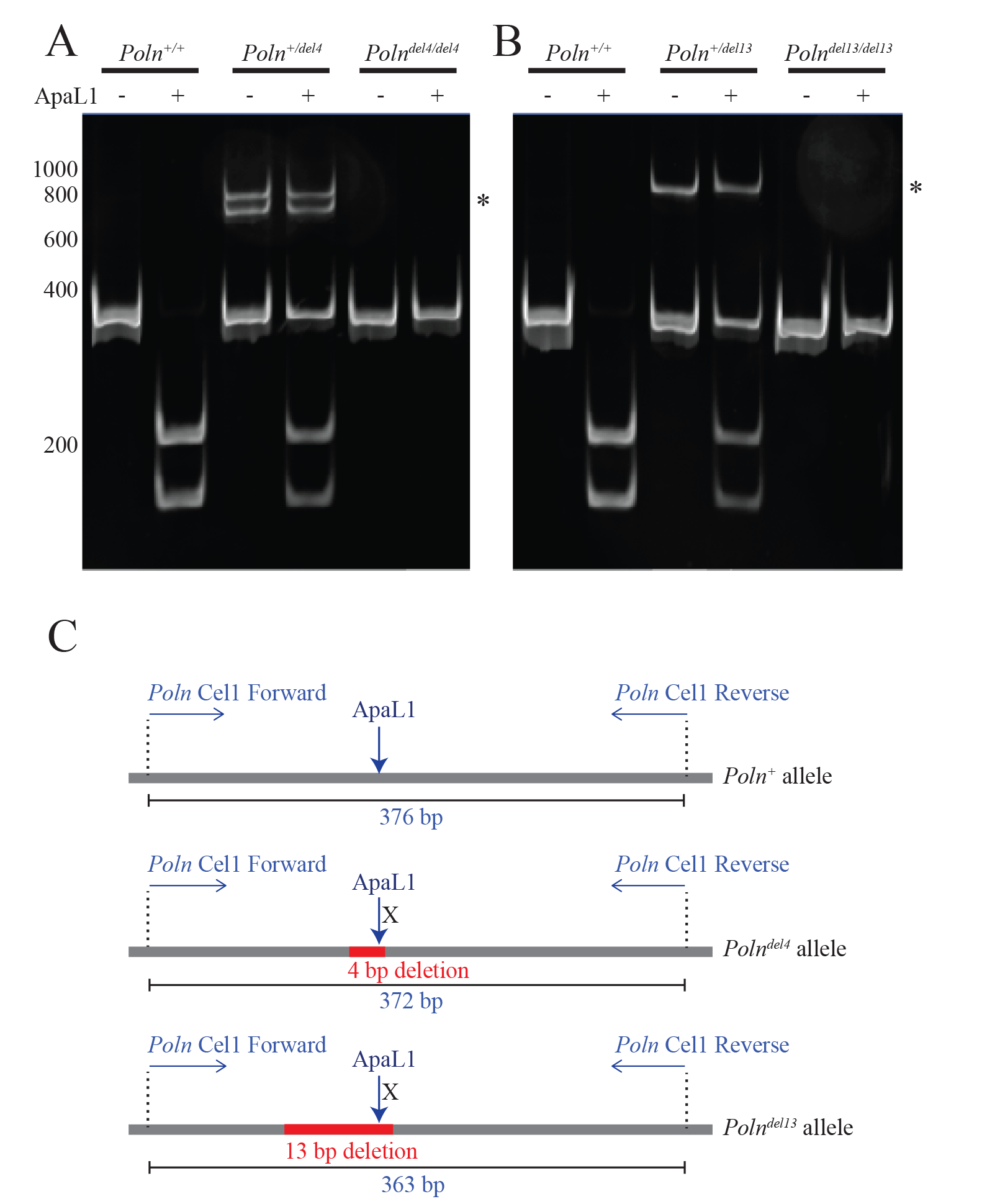

Supplement: S3 Fig — (A) Polyacrylamide gel showing typical genotype results for wild-type and Polndel4 alleles with Poln Cel1 primers. The wild-type allele is a 376-bp product and the Polndel4 allele product is indistinguishable in size from the wild-type allele. Digestion of the wild-type Poln allele with the restriction enzyme ApaL1 yields 205-bp and 171-bp PCR products. Due to ablation of the ApaL1 restriction site, the Polndel4 allele product is resistant to digestion. *Denotes heteroduplex product. (B) Polyacrylamide gel showing typical genotype results for wild-type and Polndel13 alleles with Poln Cel1 primers. The wild-type allele is a 376-bp product and the Polndel13 allele product is indistinguishable in size from the wild-type allele. The Polndel13 allele product is also resistant to ApaL1 digestion. *Denotes heteroduplex product. (C) Diagram of the ZFN targeted mouse Poln allele, with the wild-type (Poln+) allele shown at the top. The blue arrow indicates the ApaL1 restriction site. The middle diagram represents the Polndel4 targeted allele. The red bar denotes the location of the 4-bp deletion. The bottom diagram represents the Polndel13 targeted allele. The red bar denotes the location of the 13-bp deletion. Locations of Poln Cel1 primers and expected full-length product sizes are shown for each allele. (TIF) [file pgen.1006818.s003.tif]

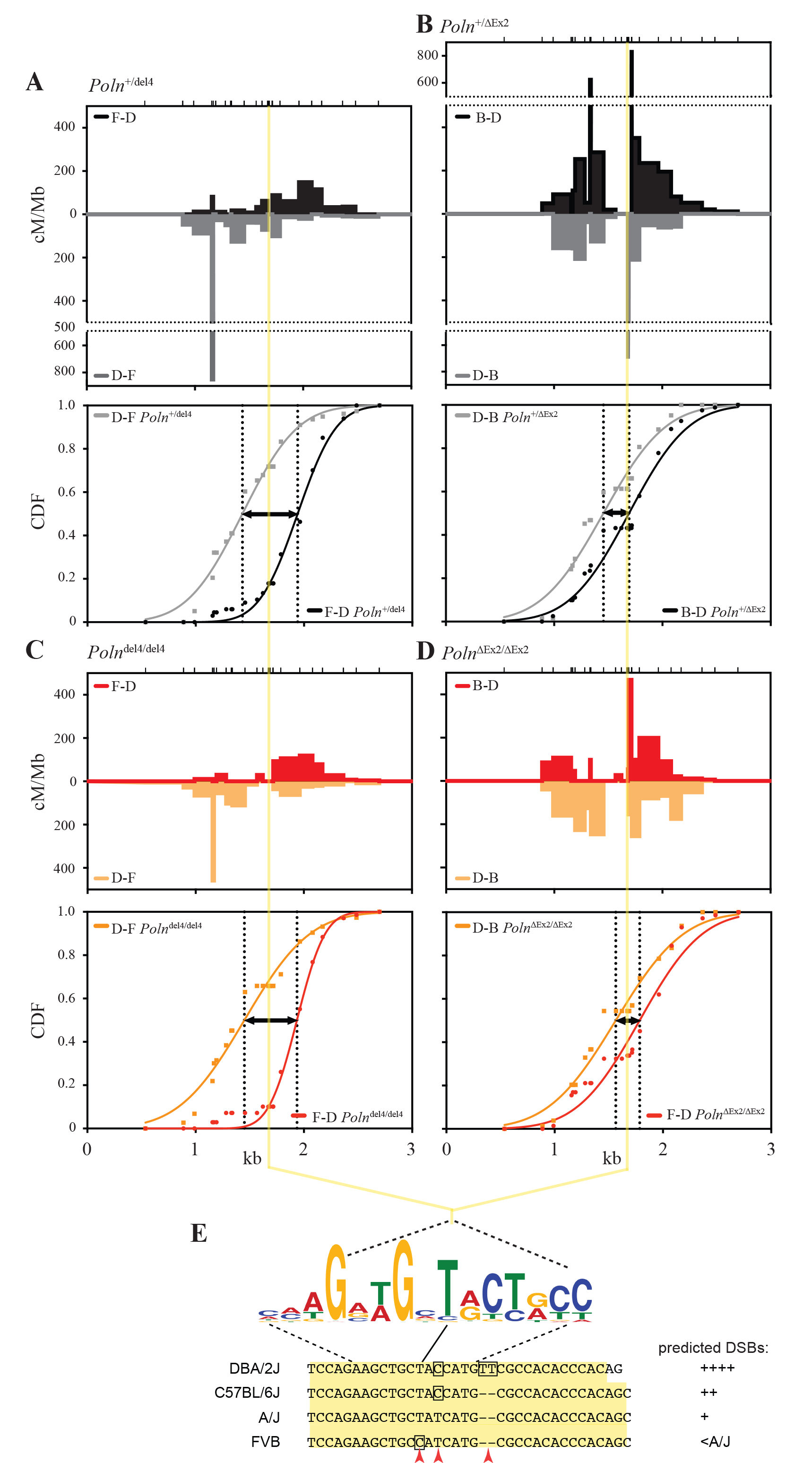

Supplement: S4 Fig — (A) Top, crossover breakpoint maps from FVB/NCrl (F) x DBA/2J (D) in the F-to-D (black) and D-to-F (gray) orientations for Poln+/del4. Bottom, cumulative distribution fraction (CDF) of crossover breakpoints. The dashed vertical lines represent the average midpoint of crossover breakpoints in each orientation. Note that the midpoints in the F-to-D and D-to-F orientation are shifted relative to each other, indicating strong reciprocal crossover asymmetry. The asymmetric distribution in crossover breakpoints is interpreted to reflect biased DSB formation in favor of one of the parental chromosomes, in this case D. (B) Top, crossover breakpoint maps from C57BL6/J (B) x D in the B-to-D (black) and D-to-B (gray) orientations for Poln+/ΔEx2. Bottom, CDF of crossover breakpoints showing mild reciprocal crossover asymmetry. (C) Similar to (A): Top, crossover breakpoint maps in the F-to-D (red) and D-to-F (orange) orientations for Polndel4/del4. Bottom, strong reciprocal crossover asymmetry is observed in the F x D background. (D) Similar to (B): Top, crossover breakpoint maps in the B-to-D (red) and D-to-B (orange) for PolnΔEx2/ΔEx2. Bottom, mild reciprocal crossover asymmetry is observed in the B x D background. (E) The binding of a meiosis-specific zinc finger protein, PRDM9, dictates the location of most meiotic DSBs in the mouse. There is a PRDM9 binding motif at the center of the A3 locus [26]. The consensus PRDM9 binding motif derived by Brick et al. [52] is also indicated. The binding motif sequence differs between strain backgrounds, which affects the affinity of PRDM9 binding [26] and likely the frequency of meiotic DSBs. The yellow bar and yellow shading represent the predicted PRDM9 binding site. Red arrowheads indicate polymorphisms implicated in differential PRDM9 binding. The PRDM9 binding motif in FVB/NCrl is predicted to have lower affinity than the motif in the A/J background. It was previously shown that in A/J x DBA/2J F1 hybrids, the A3 hotspot has a stro [file pgen.1006818.s004.tif]

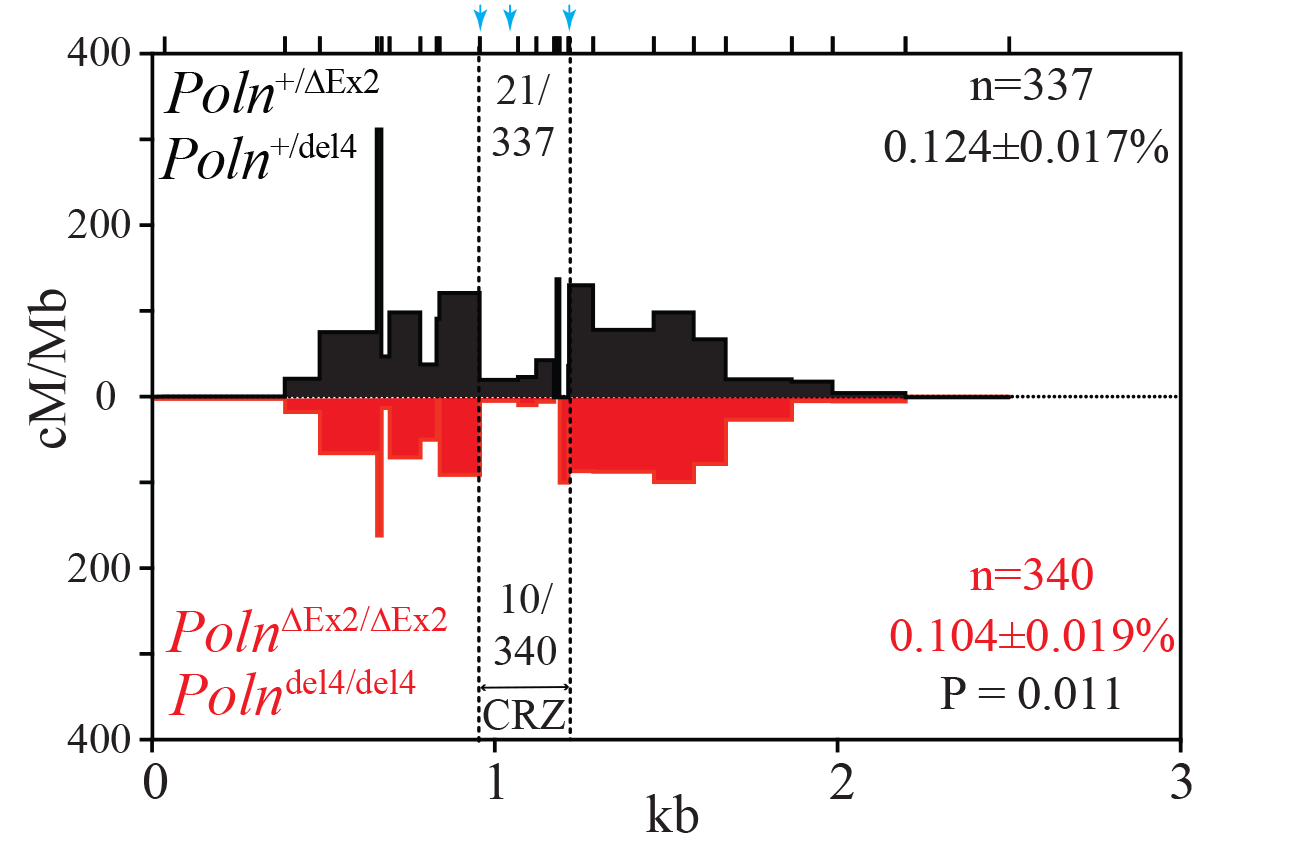

Supplement: S5 Fig — Total crossover breakpoints found in 337 Poln heterozygote controls (157 Poln+/del4 and 180 Poln+/ΔEx2) and 340 Poln knockouts (155 Polndel4/del4 and 185 PolnΔEx2/ΔEx2) are shown. Numbers of crossovers examined, Poisson-adjusted frequencies (± SD), and P-values (Fisher’s exact test) are indicated. Ticks represent positions of the tested polymorphisms. Arrows, insertion/deletion polymorphisms. (TIF) [file pgen.1006818.s005.tif]

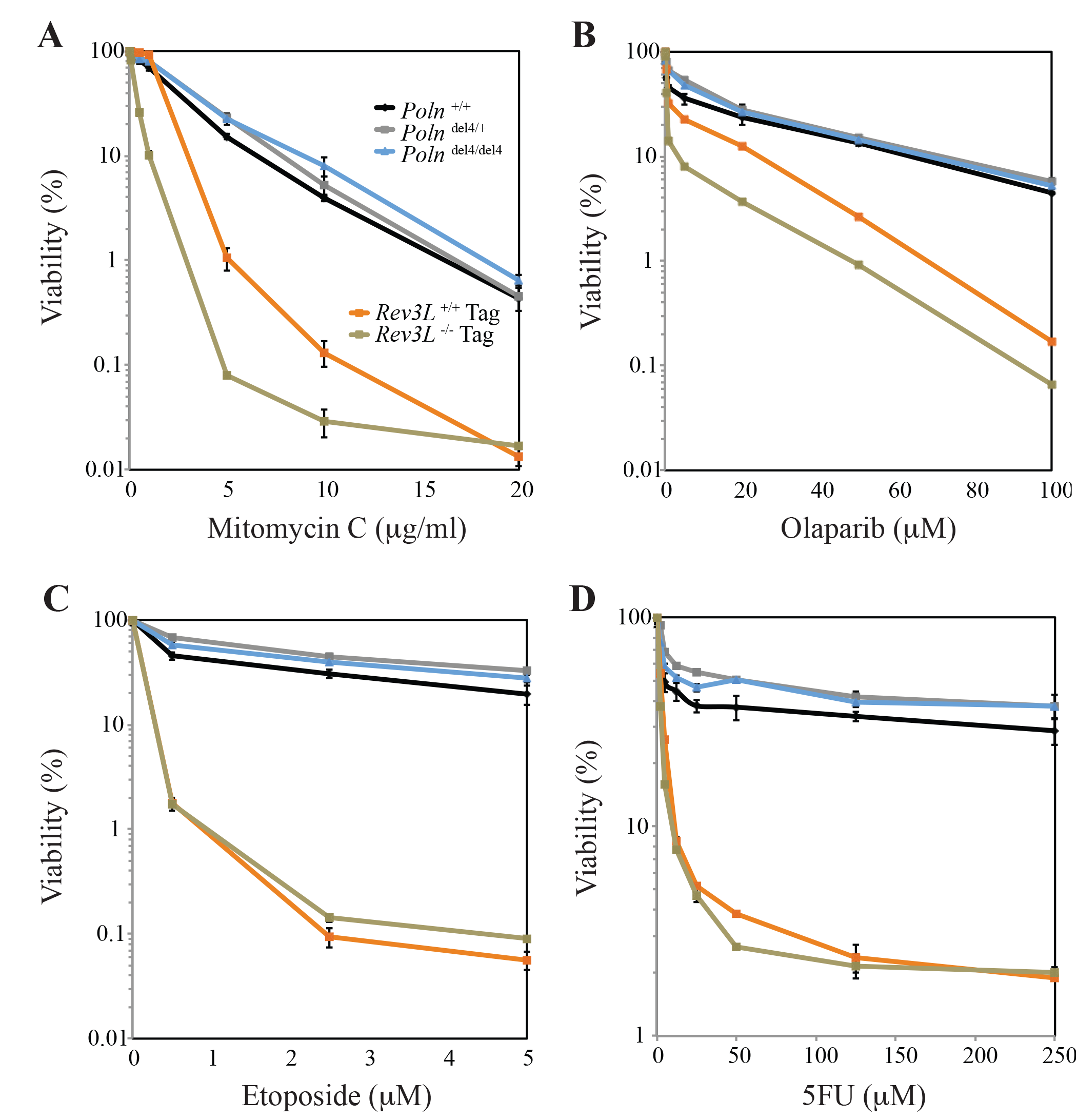

Supplement: S6 Fig — Cells were exposed to indicated doses of mitomycin C for 72 hr (A), olaparib for 72 hr (B), etoposide for 72 hr (C) and 5-FU for 72 hr (D). Poln+/+ primary MEFs (circles), Poln+/del4 primary MEFs (triangles), Polndel4/del4 primary MEFs (cross), Rev3L+/+;p53−/− SV40 Tag-immortalized MEFs (lozenge) and Rev3L-/-;p53−/− Tag-immortalized MEFs (square). Viability was determined by measuring ATP content as described in Materials and Methods. The mean of three separately plated and treated experiments is shown, with SD indicated by error bars. (TIF) [file pgen.1006818.s006.tif]

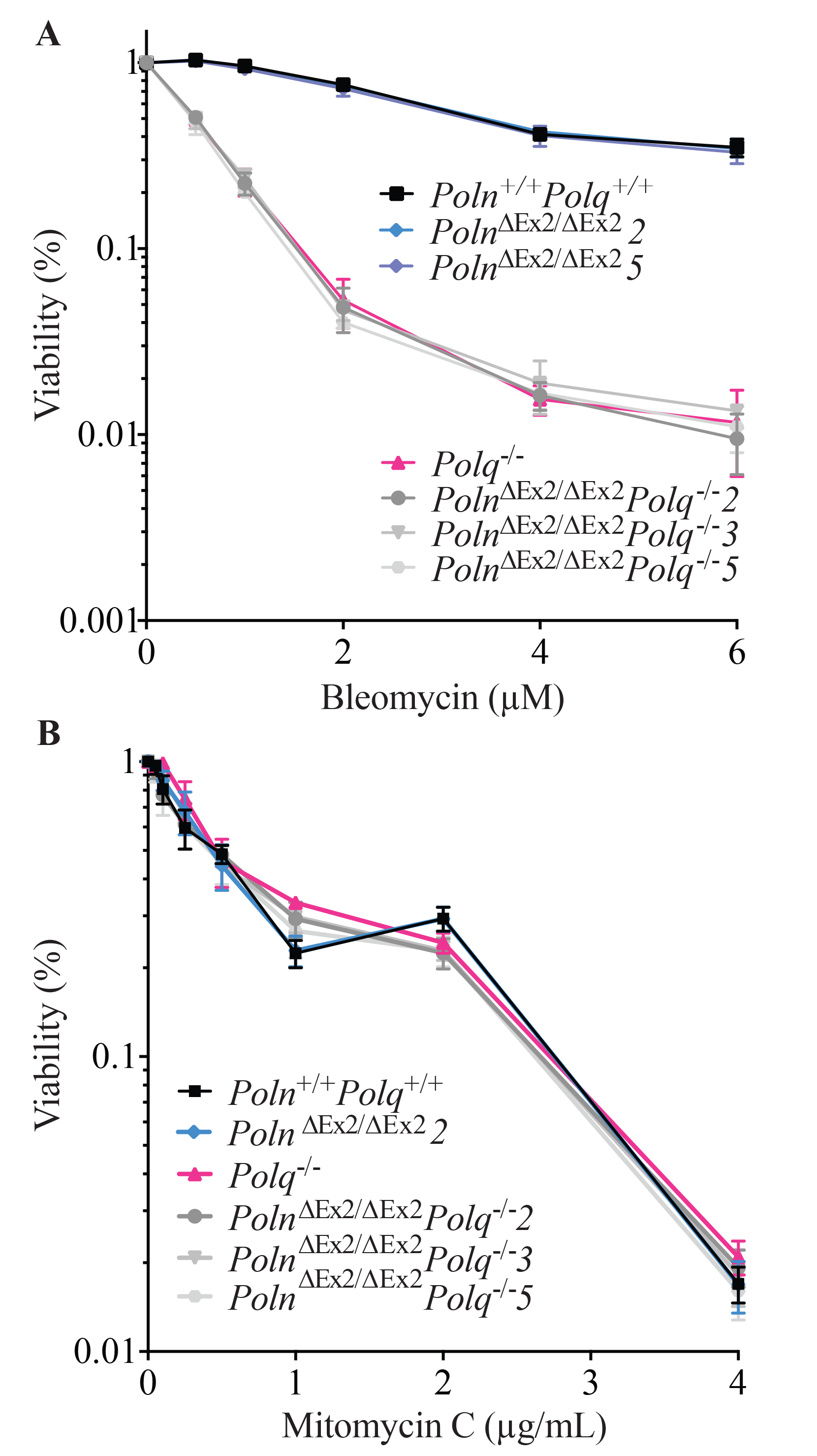

Supplement: S7 Fig — MEFs were exposed to indicated doses of bleomycin for 24 hr and incubated for 72 hr (A) and mitomycin C for 48 hr (B). Poln+/+ Polq+/+: wild-type, PolnΔEx2/ΔEx22 and PolnΔEx2/ΔEx25: individual Poln knockout, Polq-/-: Polq knockout, PolnΔEx2/ΔEx2 Polq-/- 2,3, and 5: individual Poln Polq double knockout. All MEFs were SV40 Tag-immortalized. Viability was determined by measuring ATP content as described in Materials and Methods. The mean of three separately plated and treated experiments is shown, with SD indicated by error bars. (TIF) [file pgen.1006818.s007.tif]

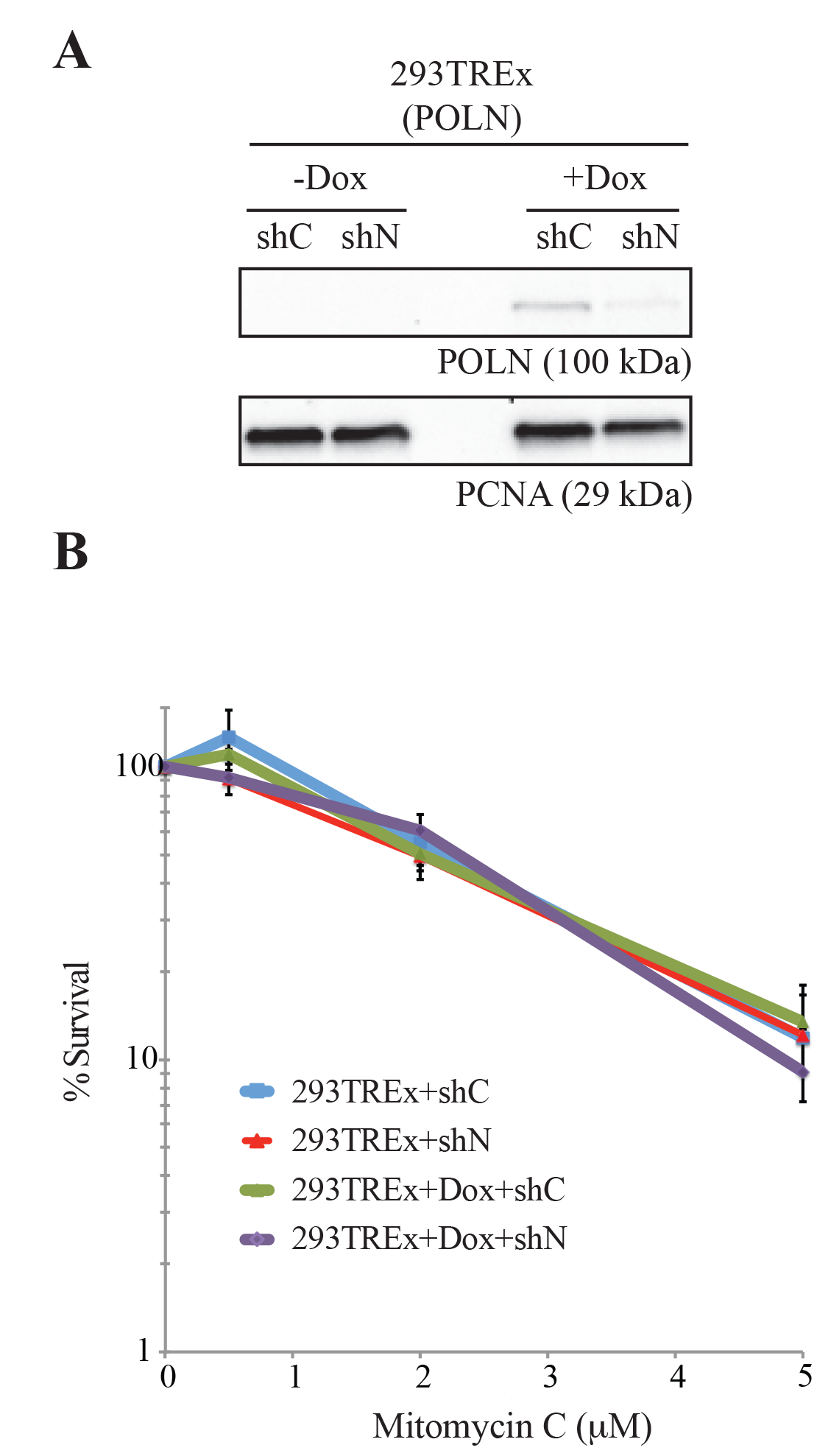

Supplement: S8 Fig — (A) Upper panel: immunoblot showing efficacy of shRNA-mediated knockdown of POLN (shN) in 293T-REx doxycycline inducible POLN cells. shC served as a negative control and PCNA as loading control. Monoclonal anti-pol ν antibody (Mab#40) recognized overexpressed pol ν but not endogenous pol ν. (B) Cell survival determined by using clonogenic survival assays. The mean of two independent experiments is shown, with SE indicated by error bars. (TIF) [file pgen.1006818.s008.tif]

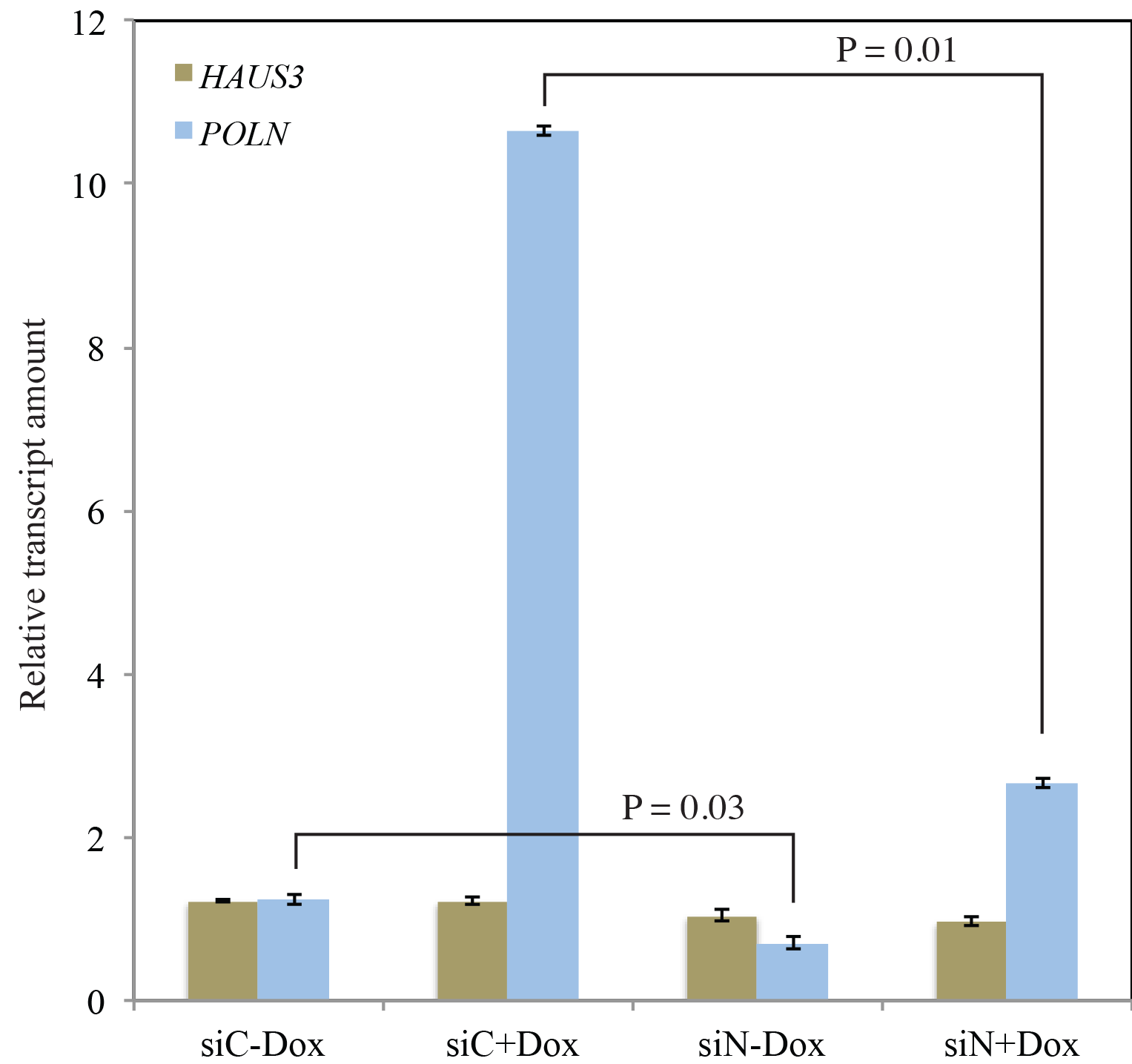

Supplement: S9 Fig — The efficacy of siRNA-mediated knockdown of POLN (siN) in 293T-REx doxycycline inducible POLN cells. The TaqMan primers spanned across adjacent exons of the human gene as described [21]. siC served as a negative control. HAUS3 was analyzed simultaneously. Note that full-length POLN is not appreciably expressed in 293T cells, although partial transcripts representing portions of the mRNA can be detected [21]. To evaluate the extent of the reduction, a paired t-test was performed. (TIF) [file pgen.1006818.s009.tif]
