# Supplementary material for: Analysis of DNA polymerase ν function in meiotic recombination, immunoglobulin class-switching, and DNA damage tolerance
Source: PLoS Genet. 2017 Jun 1;13(6):e1006818. doi: 10.1371/journal.pgen.1006818 (PMC5472330; doi:10.1371/journal.pgen.1006818)

**S2 Table.** **Assay for CSR from IgM to IgG1.** (A) Numbers show the percentage of the population that is IgG1 positive. (B) Representative flow cytometric plots of triplicate cultures from one mouse is displayed. Naïve splenic B cells from wild-type (WT) and *Poln*^∆Ex2/∆Ex2^ mice were stimulated with LPS and IL-4 and either mock-treated or treated with the DNA-PKcs inhibitor NU7026. After 72 hours, cells were assayed for IgG1 expression (y-axis) by flow cytometry. Forward scatter (FSC, x-axis) and propidium iodide staining (gate not shown) was used for cell viability. Numbers in boxes show the percentage of the population that is expressing IgG1. (C) No significant difference between wild-type and *Poln* *^ΔEx2^*^/^*^ΔEx2^* in the presence or absence of NU7026 was determined by student's t-test.

**A**

| **Untreated** |  | sample A | sample B | sample C | Average |
| --- | --- | --- | --- | --- | --- |
| wild-type | Mouse #1 | 6.1 | 5.8 | 6.3 | 6.1 |
|  | Mouse #2 | 6.8 | 6.5 | 6.9 | 6.7 |
|  | Mouse #3 | 6.1 | 5.3 | 5.4 | 5.6 |
|  |  |  |  | **TOTAL:** | **6.1 ± 0.6** |
| *Poln* *^ΔEx2^*^/^*^ΔEx2^* | Mouse #1 | 5.6 | 5.4 | 5.4 | 5.5 |
|  | Mouse #2 | 6.6 | 6.1 | 6.2 | 6.3 |
|  | Mouse #3 | 6.7 | 6.5 | 6.4 | 6.5 |
|  |  |  |  | **TOTAL:** | **6.1 ± 0.5** |
| **NU7026** |  | sample A | sample B | sample C |  |
| wild-type | Mouse #1 | 12.4 | 12.5 | 11.9 | 12.3 |
|  | Mouse #2 | 14.3 | 14.1 | 13.9 | 14.1 |
|  | Mouse #3 | 8.9 | 8.4 | 8.8 | 8.7 |
|  |  |  |  | **TOTAL:** | **11.7 ± 2.8** |
| *Poln* *^ΔEx2^*^/^*^ΔEx2^* | Mouse #1 | 12.9 | 12.8 | 12.8 | 12.8 |
|  | Mouse #2 | 12.8 | 13.1 | 13.3 | 13.1 |
|  | Mouse #3 | 12.1 | 11.2 | 11.8 | 11.7 |
|  |  |  |  | **TOTAL:** | **12.5 ± 0.7** |

**B** **C**


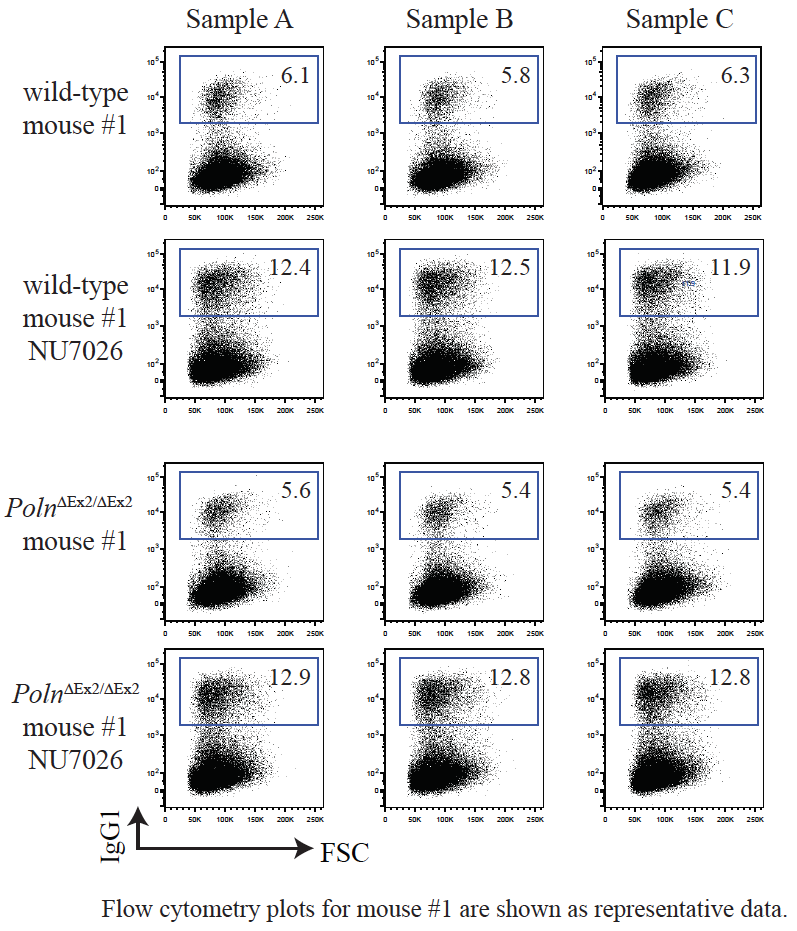

Supplement: S2 Table — (DOCX) [file pgen.1006818.s011.docx]
